# Pharmaco-Technological Characterization, Structural Analysis, and Toxicological Evaluation of the Novel Polyene Antibiotic Roseofungin for Drug Development

**DOI:** 10.3390/pharmaceutics17040430

**Published:** 2025-03-27

**Authors:** Amankeldy Sadanov, Dmitriy Berillo, Assya Bagimbayeva, Gul Baimakhanova, Liliya N. Ibragimova, Iliyas Raikhanovich Kulmaganbetov, Farida Nurmaganbetova, Gulbany Sarsenbaeva, Saltanat Orazymbet, Baiken Baimakhanova, Olga Lakh, Diana Tleubekova, Gulnara T. Dzhakibaeva, Tulegen Mussaldinov

**Affiliations:** 1 Scientific Production Center for Microbiology and Virology LLP, Almaty 050010, Kazakhstan; 2 Department of Chemistry, M. Kozybayev North-Kazakhstan University, Zhumabaeva Str., 114, Petropavlovsk 150000, Kazakhstan; 3 Department of Pharmaceutical and Toxicological Chemistry, JSC Asfendiyarov Kazakh National Medical University, Almaty 050012, Kazakhstan; 4 Center for Pharmacy and Pharmacology of the Science and Technology Park, JSC Asfendiyarov Kazakh National Medical University, Almaty 050012, Kazakhstan

**Keywords:** roseofungin, antibiotic, pharmaco-technological parameters, FTIR, HPLC, NMR, size distribution, standardization, impurities

## Abstract

**Background/Objectives:** pentane polyene antibiotic Roseofungin produced by actinomycetes possessing wide range of antimicrobial activity. **Methods:** The structure of novel polyene antibiotic Roseofungin was confirmed through FTIR, H^1^ nuclear magnetic resonance, and high-performance liquid chromatography analysis with a mass detector. The substance pharmaco-technological parameters were evaluated. Additionally, the in silico modelling of various physicochemical parameters and biological activities was performed using validated open-access software tools such as ProTox3, SwissADME, and ADMET SAR1. The evaluation of its toxicological profile was also investigated in vivo. **Results:** The Roseofungin exhibits potential risks in certain categories, including immunotoxicity, respiratory toxicity, and nephrotoxicity, as predicted in silico. However, Roseofungin shows a relatively safe profile with regard to hepatotoxicity, neurotoxicity, and mutagenicity, along with lower risks of carcinogenicity and cytotoxicity in silico. The analysis of body weight dynamics after Roseofungin exposure in mice revealed no statistically significant differences among the experimental groups. Similarly, in the absolute or relative weights of internal organs across the experimental groups after Roseofungin treatment, no significant differences were observed in vivo. Roseofungin appears as a light-yellow hygroscopic powder with a specific odour, possessing the ability to settle and classified as a light powder. The particles are lamellar crystals ranging in size from 3 μm to 6 μm, and the molecules generate electrostatic tension between themselves. The pharmaco-technological parameters of Roseofungin were comprehensively studied. **Conclusions:** The experimental data obtained provide a foundation for further pharmaceutical development of new drugs containing the original Roseofungin.

## 1. Introduction

The issue of global antibiotic resistance is quite acute. Carbapenem-resistant *Acinetobacter baumannii* (CRAB) has become a significant global pathogen, with few available treatment options. In fact, no new class of antibiotics effective against *A. baumannii* has been introduced to patients in over 50 years [1]. The WHO annually emphasizes this problem as relevant and has prioritized the search for new chemotherapeutic agents with antibacterial activity that have not formed microorganismal resistance [2]. Several recent reviews have addressed the growing global public health threat posed by multi-resistant bacteria, which are now widely recognized as emerging contaminants. These reviews discuss various strategies to combat antibiotic resistance, such as optimizing dosage and using combined formulations, which have proven effective in mitigating the negative impacts of antibiotic resistance [3].

Chronic infections are often linked to polymicrobial biofilms, which are resistant to standard antimicrobial treatments. The interactions within the microbial communities can alter antibiotic effectiveness, leading to treatment failure [4]. A recent review in *Nature* explores compounds that could inspire the development of urgently needed new antibacterial agents. The review examines the complex mechanisms evolved to effectively target bacteria, such as covalent binders, resistance inhibitors, self-promoted entry compounds, resistance-evading molecules, and prodrugs. By understanding the intricate nature of the most effective antimicrobial compounds, the review offers a roadmap for creating novel agents to combat antimicrobial resistance, emphasizing the potential of mining new natural products and designing equally advanced antibiotics [5]. The review summarizes that polyenes are small pharmaceutical molecules known to induce various effects on lipid membranes and biological cells. Numerous studies have aimed to uncover the mechanisms behind the interaction between these molecules and membranes, providing valuable insights. Despite their significant host toxicity, polyenes continue to be researched due to their high antifungal efficacy and the low occurrence of resistance, making them important candidates for further study [6]. Caffrey’s research group explored the potential of synthetic biology to aid the production of new antifungal agents by investigating three classes of polyene glycosyltransferases. These include enzymes that extend glycosylation by forming disaccharide-containing polyenes, glycosyltransferases responsible for adding L-digitoxose sugars to nystatin A3 and selvamicin, and mycosaminyltransferases that introduce the primary aminodeoxysugar. Increasing the level of glycosylation in polyene antibiotics has been shown to reduce their toxicity [7].

According to Kotler-Brajtburg et al., polyene antibiotics can be categorized into different groups based on their chemical structure. Group I antibiotics were found to induce potassium leakage, leading to cell death or hemolysis at comparable concentrations of the polyenes used. The antibiotics in this group include the following: Trienins; Tetranes, such as *pimaricin* and *etruscomycin*; Pentanes, including filipin and chainin; a single hexane (dermostatin); and *Lymphosarcin* [8,9]. Moreover, the release of potassium from *C. guilliermondii* cells after *Roseofungin* treatment is due to the inhibition of (Na^+^, K^+^)-ATPase, and an increase in the permeability of the plasma membrane was observed [9].

The rapid spread and growth of many pathogenic fungi, combined with their resistance to common antimicrobial treatments, highlight the need for effective and reliable antifungal therapies. Zotchev, S.B. summarized the data on polyene macrolide antibiotics, which have been the most successful antifungal agents, known for their strong fungicidal properties, wide range of action, and the relatively low occurrence of resistance among fungal pathogens [10]. Tevyashova et al. investigated the antifungal activity of N-(2-aminoethyl)amide of AmB (amphamide, 6) in vitro, demonstrating that it had superior antifungal activity compared to the parent compound, AmB. Preclinical studies in mice further showed that amphamide (compound 6) exhibited significantly lower acute toxicity and greater antifungal efficacy in vivo. This discovery positions amphamide as a promising candidate for the next generation of polyene antibiotics, warranting further preclinical and clinical evaluation [11].

In this regard, of particular scientific and practical interest is the domestic carbonyl-conjugated pentane polyene antibiotic Roseofungin, which was developed at LLP Scientific Production Center Microbiology and Virology [12,13]. Roseofungin is intended for external use to treat fungal lesions of the feet and skin [14,15,16]. The characterization of *Roseofungin* as a pentane antibiotic of the subgroup of carbonyl-conjugated pentanes was previously performed using physicochemical methods including HPLC, the mass spectrometry of fragments, and NMR [17,18,19]. Vetlugina et al. obtained the substance [20]. Roseofungin possesses strong antifungal activity, suppressing the causative agents of superficial and deep mycoses, including trichophytosis, microsporia, favus, candidiasis, cryptococcosis, sporotrichosis, chromomycosis, aspergillosis, etc., at concentrations ranging from 0.5 to 12.5 μg/mL [21,22]. Activity against *Candida albicans* and non-*albicans* species is in the range of 1.11–1.65 μg/mL. Despite the fact that most polyene antibiotics used in medicine predominately exhibit inhibitory activity against yeast fungi, Roseofungin is also highly active against dermatophytes: its minimum inhibitory concentration (MIC) against *Trichophyton mentagrophytes* var. *gypseum* is 0.53 μg/mL, against *Microsporum lanosum* is 0.78 μg/mL, and against *Trichophyton rubrum* is 1.11 μg/mL. Roseofungin is highly active against the favus pathogen *Achorion schoenleinii*, with an MIC value of 0.6 μg/mL [23,24]. The MICs of Roseofungin against six species of fungi that cause trichophytosis (*Trichophyton tonsurans*, *T. violaceum*, *T. soudanense*, *T. simii*, *T. ajelloi*, *T. vanbreuseghemii*) varies from 0.78 to 3.12 μg/mL; against six species of microsporia pathogens (*Microsporum canis*, *M. audouinii*, *M. ferrugineum*, *M. cookei*, *M. gypseum*, *M. vanbreuseghemii*), 1.56–3.12 mg/mL; and the activity of Roseofungin against *Epidermophyton floccosum* is equalled to 3.12 μg/mL [25]. The antibiotic also shows strong activity against pathogens of tropical mycoses. The MICs against *Cryptococcus neoformans* (the causative agent of cryptococcosis), *Sporotrichum schenckii* (the causative agent of sporotrichosis), and *Hormodendrum* spp. (the causative agent of chromomycosis) are 4.69 mg/mL, 7.29 mg/mL, and 5.21 mg/mL, respectively [24,25]. It has been established that Roseofungin has comparative-to-model active pharmaceutical substance activity against moulds. Additionally, the activity of *Roseofungin* against fungi of the genus *Aspergillus*, which cause superficial mycoses, is in the range of 4.6–8.33 mg/mL, which is more efficient compared to widely commercially available drugs [19].

Comparative studies [25] have shown that the spectrum of antifungal activity of *Roseofungin* is much wider than that of other polyene antibiotics (nystatin, amphotericin B, levorin) used in medical practice. Compared to other representatives from the group of polyene antibiotics (mediomycins, clethramycin), *Roseofungin* shows higher activity against pathogenic fungi and is more stable and less toxic [20,21].

When examining the mechanism of action of *Roseofungin* against *Candida guilliermondii* cells, the effects of this antibiotic on the release of potassium and phosphorus from cells was shown [26,27]. It was found that the yield of potassium and phosphorus ions depends on the concentration of the antibiotic and the incubation time. Exposure to *Roseofungin* at doses above 20 mg/mL causes the cells to quickly lose their accumulated potassium, and the potassium ions are completely released from the cells at a concentration of 200 μg/mL *Roseofungin*. The release of inorganic phosphorus from the cell was recorded at *Roseofungin* doses of 40 μg/mL and higher. *Roseofungin* at 10 mg/mL concentration inhibits respiration in a suspension of fungal cells [27]. Oxygen consumption by cells treated with *Roseofungin* (40 mg/mL) sharply decreased. With an increase in the concentration of *Roseofungin* to 200 mg/mL, respiration was almost completely inhibited. The effects of *Roseofungin* on the activities of the Mg^2+^-dependent ATPase and (Na^+^, K^+^)-activated ATPase on *C. guilliermondii* plasma membranes were studied [28].

Roseofungin also has pronounced antiviral activity against influenza and parainfluenza viruses [29,30,31,32,33,34]. The preventive administration of *Roseofungin* at the dose of 25 μg per individual caused protection against lethal infection to reach 80%; however, when treating infected persons, the optimal dosage is 10 μg. An increased dose of the antibiotic had the opposite effect, and the level of protection was significantly reduced [29,30].

Studies on the virus-inhibiting activities of *Roseofungin* during chicken embryo infection with avian influenza virus (strain A/FPV/Rostok/34, H_7_N_1_) and human influenza virus (strain A/MRC-11, H_3_N_2_) showed the activity of *Roseofungin* as comparable to that of *Tamiflu* (Hoffman-LaRoche, Switzerland) and exceeded that of *Rimantadine* (NS Pharma, Russia) and *Ribavirin* (Vertex, Russia). The antiviral mechanism action of *Roseofungin* is associated with the inhibition of neuraminidase activity, which provides possible viral entry into the cell and releases viral progeny from the cell. There is evidence that *Roseofungin* has antiviral activity against other viruses, in particular, the vaccinia virus and the Rous sarcoma virus [33,34]. The purpose of this work is to study the *Roseofungin* pharmaco-technological parameters for further pharmaceutical industrial manufacturing of various pharmaceutical formulations. FTIR, ^1^H-NMRspectra, and the HPLC analysis of *Roseofungin* substance are discussed.

## 2. Materials and Methods

### 2.1. Materials

d6-DMSO (99.9% Sigma Aldrich, St. Louis, MO, USA) MQ water, acetonitrile HPLC grade SigmaAldrich was used. DMSO (99.5% Sigma Aldrich), pyridine (99.0% Sigma Aldrich), dichloromethane (99.5% Sigma Aldrich), chloroform (99.5% Sigma Aldrich), n-hexane (98% Sigma Aldrich), butanol (99.7% Sigma Aldrich), benzene (99.7% Sigma Aldrich), Witepsol^®^ W (IOI Oleo GmbH, Hamburg, Germany), solid fat (Suppocire^®^ CM, Gattefossé, Lyon, France), cocoa butter (LLP «Lotte Rachat», Almaty, Kazakhstan), and sunflower oil (TM «3 Wishes», Almaty, Kazakhstan) were used. Tween^®^ 80 (Sigma Aldrich), ethyl alcohol (96%), glycerine (99.5% Sigma Aldrich), PEG 400 (Sigma Aldrich) and PEG 1500 (Sigma Aldrich), acetic acid (99.0% Sigma Aldrich), and lanolin (99.0% BOC Sciences, Shirley, NY, USA) were used. The Active Pharmaceutical Ingredient (API) *Roseofungin* was provided by Scientific Production Center for Microbiology and Virology LLP, Almaty, Kazakhstan.

### 2.2. NMR

NMR spectra of Roseofungin in d6-DMSO ^1^H and ^13^C NMR spectra of Roseofungin were recorded in d6-DMSO using JNM-ECA Jeol 400 (JEOL Ltd., Tokyo, Japan) (frequency 399.78 and 100.53 MHz).

### 2.3. FTIR

FTIR spectra of Roseofungin was recorded from a powder using ATR-IR Fourier spectrometer Carry 660 Agilent (Agilent Technologies, Santa Clara, CA USA).

### 2.4. HPLC Analysis

HPLC analysis was carried out using Agilent 1200 series (Agilent, Waldbronn, Germany), equipped with a four-channel gradient pump, degasser, autosampler, column thermostat and diode array detector. Separation was performed on an Agilent Zorbax SB-C18 4.6 × 150 mm column, particle diameter of 5 μm. The total analysis time was set to 60 min in a gradient model (Table 1), which showed better separation than previously ascribed isocratic mode. The isocratic mode utilizes a mixture of acetonitrile 0.1% triflouroacetic acid: water 0.1% triflouroacetic acid (60:40) as an eluent and at a flow rate of 1000 μL/min. The column temperature was kept constant at 30 °C. The volume of the injected sample was 10 μL. Detection was carried out at a wavelength of 260 nm.

The foregoing indicates the great potential of this API for the treatment of a number of diseases. Development of a gel and a solution in spray form, suppositories/pessaries for vaginal use to treat candidiasis, and dosage forms for oral administration (tablets, capsules, syrups, etc.) are promising strategies.

In the pharmaceutical development of new drugs with various dosage forms, knowledge of the data relating to the API pharmaco-technological properties is necessary, which makes the selection of a rational composition (including excipients) and the optimal technology for their preparation, making future studies more oriented [35].

The physico-chemical and technological parameters of the substance were determined according to the pharmacopeial methods described in the State Pharmacopeia of the Republic of Kazakhstan (SPh RK), the European Pharmacopeia (Ph. Eur.), and the United States Pharmacopeia (USP).

### 2.5. Description of the Substance

The drug substance in appearance complied with the requirements of the SPh RK I, Vol. 2 General monograph Pharmaceutical substances, Ph. Eur. General monograph <2034> Substances for pharmaceutical use [35,36,37].

### 2.6. Odour

The drug substance odour was assessed in accordance with the procedure described in SPh RK I, Vol. 1, 2.3.4 Odour assessment, Ph. Eur. 2.3.4 Odour [35,36].

### 2.7. Particle Shape and Size

The shape and size of the drug substance particles were determined in accordance with the procedure described in SPh RK I, Vol. 1, 2.9.13 Determination of the particle size of powders by microscopy; Ph. Eur. 2.9.37 Optical microscopy; and USP <776> Optical microscopy [35,36,37]. The microstructure of the samples was determined using scanning electron microscopy (SEM; MIRA 3 LMU, Tescan, Brno, Czech Republic). A gold layer was deposited on the surface of the nonconducting samples prior to their examination by thermal spraying with the Q150R ES coating system (Quorum Technologies, East Sussex, UK).

### 2.8. Specific Optical Rotation

The specific optical rotation of the drug substance was measured in accordance with the procedure described in SPh RK I, Vol. 1, 2.2.7 Optical rotation; Ph. Eur. 2.2.7 Optical rotation; and USP <781> Optical rotation [35,36,37,38] with an MCP 51005 polarimeter (Anton Paar, GmbH, Graz, Austria). The specific optical rotations were measured in pyridine, methanol (0.25% solutions) and dioxane (1% solution).

### 2.9. Melting Point

The melting point of the drug substance was determined in accordance with the procedure described in SPh RK I, Vol. 1, 2.2.14 Melting point—capillary method; Ph. Eur. Melting point—capillary method; and USP <741> Melting range or temperature [35,36,37,38] with a KSP1 series Melting Point Metre (A. Kruss Optronic, Hamburg, Germany).

### 2.10. Solubility

The solubility of the drug substance in various solvents was assessed in accordance with the procedure described in SPh RK I, Vol. 1, 1.4 Monographs; Ph. Eur. 1.4. Monographs; and USP Section 5 Monograph components, 5.30. Description and Solubility [35,36,37,38]. The following solvents and excipients necessary for the pharmaceutical development and standardization of the drug substance and drugs containing this substance were examined: DMSO, Tween-80, glycerine, lanoline, polyethylene glycol (PEG) 400, PEG 1500, acetic acid, 96% ethyl alcohol, purified water, sunflower oil, cocoa butter, hard fat, Witepsol (grades H, W, S, E), benzene, water-saturated butanol, n-hexane, chloroform, pyridine, and dichloromethane. Analytical-grade and reagent-grade solvents and excipients of pharmacopeial quality were used.

### 2.11. Fractional Composition

The fractional composition of the drug substance was studied in accordance with the procedure described in Ph. Eur 2.9.31 Particle size analysis by laser light diffraction [35,36,37,38], and the particle size was determined by dynamic light scattering on a Zetasizer Nano ZS submicron particle size analyser (Malvern Instruments Ltd., Worcestershire, UK). Ethyl alcohol (96%) was used as the liquid phase.

### 2.12. Bulk and Tapped Volume and Density

The bulk and tapped volumes of the drug substance were determined in accordance with the procedure described in SPh RK I, Vol. 1, 2.9.34 Bulk density and tapped density and Ph. Eur. and USP <616> Bulk density and tapped density [35,36,37,38], using a JV 2000 Bulk Density Tester (COPLEY, Nottingham, UK).

The true density, relative density, and porosity of the APS Roseofungin were calculated based on the data:

(1) $$P_{t}=\frac{m*\rho_{B}}{m+G-F}=\frac{5.0045\cdot 0.9982}{5.0045+152.5948-153.4147}=\frac{4.9955}{4.1846}=1.1938\;g/{\mathrm{cm}}^{3}$$

(2) $$P_{rel}=\frac{\;P_{0}}{\;P_{y}}\times 100=\frac{0.36}{1.1938}\times 100=30.16\%$$

(3) $$Pr=\left(1-\frac{P_{1250}}{P_{t}}\right)\times 100=\left(1-\frac{0.51}{1.1938}\right)\times 100=57.28\%$$

### 2.13. Carr Compressibility Index and Hausner Ratio

The Carr compressibility index and Hausner ratio of the drug substance were evaluated in accordance with the procedure described in SPh RK I, Vol. 1, 2.9.36 Powder flow and USP <1174> Powder flow [35,36,37], using a JV 2000 Bulk Density Tester (Pharmatron AG|Uttigenstrasse 28|CH-3600 Thun|Switzerland). The flowability of the drug substance was determined by comparing the Compressibility index and Hausner ratio in accordance with that in Table 2.

The parameters under study were calculated by the following formulae:

(4) $$Hausner\;ratio=\frac{V_{0}}{V_{1250}}$$

(5) $$\mathrm{or}\;Hausner\;ratio=\frac{P_{1250}}{P_{0}}$$

(6) $$Carr\;compressibility\;index=\frac{V_{0}-V_{1250}}{V_{0}}\times 100$$

(7) $$\mathrm{or}\;Carr\;compressibility\;index=\frac{P_{1250}-P_{0}}{P_{0}}\times 100,\;$$

where V_0_ is the bulk volume of the material (mL);

V_1250_ is the tapped volume of the material after 1250 taps (mL);

P_0_ is the bulk density of the material (g/mL);

P_1250_ is the tapped density of the material after 1250 taps (g/mL).

### 2.14. Flowability and Angle of Repose

The flowability and angle of repose of the drug substance were evaluated in accordance with the procedure described in SPh RK I, Vol. 1, 2.9.16 Flowability; Ph. Eur. 2.9.36 Powder flow; and USP <1174> Powder flow [35,36,37,38], using a PTG S4 automated powder and granule flow analyser (Pharma Test Apparatebau AG, Hainburg, Germany).

### 2.15. Loss on Drying

A total of 1.0 g of the drug substance was dried in a vacuum drying chamber at a temperature of 60 °C and a pressure of 0.7 kPa for 3 h. The loss on drying was determined in accordance with the procedure described in SPh RK I, Vol. 1, 2.2.32 Loss on drying; Ph. Eur. 2.2.32 Loss on drying; and USP <731> Loss on drying [35,36,37], using a VDL 23 vacuum drying chamber (Binder, Tuttlingen, Germany).

### 2.16. Wettability

Since the powders cannot form a completely flat surface, the powder is usually compacted like a disc in an attempt to make the surface smoother. A drop of liquid with a given volume is placed on the disc, which allows for direct measurements of the contact angle using a goniometer equipped with an ocular protractor. The wettability was assessed in accordance with the procedure described in BP 2.9.45 Wettability of porous solids including powders [35,36,37].

### 2.17. Hygroscopicity

The hygroscopicity of the drug substance was determined in accordance with SPh RK I, Vol. 1, 5.11 Characters section in monographs; and Ph. Eur. 5.11. Characters section in monographs [35,36,37], which are in line with the procedures described below. Using an SPS602F analytical balance (Ohaus Pioneer, Parsippany, NJ, USA) and a DLBA-A-BA-e-16133 electronic moisture analyser (KERN, Balingen, Germany), interpretation of the data obtained was in accordance with that in Table 3.

Method 1: Purified water, *P*, was poured into a desiccator simulating 100% moisture content. Then, a grid was set up, and 50 g of the test substance was placed on the grid in a porcelain cup. The desiccator was installed in a climate chamber with a temperature of 25 ± 1 °C, and the residual moisture of the powder was measured at predetermined times: 0, 2, 4, 8, 12, 18, and 24 h. Before each determination, the change in mass was calculated by the weight method.

Method 2: A saturated solution of sodium chloride, *R*, was poured into the desiccator simulating 75% moisture content. Then, a grid was set up, and 50 g of the test substance was placed on the grid in a porcelain cup. The desiccator was installed in a climate chamber with a temperature of 25 ± 1 °C, and the residual powder moisture was measured using a moisture metre at predetermined times: 0, 2, 4, 8, 12, 18, and 24 h. Before each determination, the change in mass was calculated by the weight method.

### 2.18. Density

The density of the drug substance was measured in accordance with SPh RK I, Vol. 1, 2.2.42 Density of solids and Ph. Eur. 2.2.42 Density of solids [35,36,37,38] with the procedure described below using an SPS602F analytical balance (Ohaus Pioneer, Parsippany, NJ, USA) and certified class A laboratory ware.

Method: The drug substance (5.0 g) was placed into a 100 mL pycnometer that filled with *purified water*, *P*, to 2/3 of the volume, kept in a water bath for 1.5–2 h, and periodically stirred to completely remove the air. After cooling to a temperature of 20 °C, the volume was filled to the mark with *purified water*, *P*. The mass of the pycnometer filled with *purified water*, *P*, was previously determined.

The true density (P_t_) was calculated by the formula:

(8) $$P_{t}=\frac{m*\rho_{w}}{m+G-F}\;g/{\mathrm{cm}}^{3}$$

where m is the mass of the absolutely dry substance (g);

G is the mass of the pycnometer filled with water (g);

F is the mass of the pycnometer filled with water and drug substance (g);

$\rho_{w}$ is the density of water (0.9982 g/cm^3^).

### 2.19. Relative Density

The relative density of the drug substance was calculated in accordance with SPh RK I, Vol. 1, 2.2.42 Density of solids and Ph. Eur. 2.2.42 Density of solids [35,36,37,38] according to the formula below:

(9) $$P_{rel}=\frac{P_{0}}{\;P_{t}}*100\%,$$

where P_0_ is the bulk density (g/cm^3^) and P_t_ is the true density (g/cm^3^).

### 2.20. Porosity

The porosity of the drug substance was calculated in accordance with SPh RK I, Vol. 1, 2.2.42 Density of solids and Ph. Eur. 2.2.42 Density of solids [35,36,37,38].

(10) $$Pr=\left(1-\frac{P_{1250}}{P_{t}}\right)*100,$$

where P_1250_ is the tapped bulk density (g/cm^3^) and P_t_ is the true density (g/cm^3^).

Statistical processing of the results was carried out by a suitable mathematical statistic method as described in the State Pharmacopeia (relative standard deviation and confidence interval using Student’s test with a probability of *p* < 0.05) [35,36,37]. Minitab 19 (Minitab, LLC, State College, PA, USA) and Excel (Microsoft Corporation, Washington, DC, USA) were used for calculations.

### 2.21. In Silico Modelling

#### 2.21.1. ADME Prediction for the Investigation of Pharmacokinetic Parameters of API *Roseofungin* Was Carried Out Using Open Free Software Previously Used

To facilitate the analysis of the pharmacokinetic characteristics of Roseofungin, free tools such as SwissADME http://www.swissadme.ch/ (accessed on 20 December 2024) [39] were used.

ADMETlab 2.0, and AdmetSAR are available at https://lmmd.ecust.edu.cn/admetsar2 (accessed on 20 December 2024), http://admet.scbdd.com/ and http://lmmd.ecust.edu.cn/admetsar1 (accessed on 22 December 2024), respectively. These platforms were used to evaluate the overall physicochemical parameters, drug-likeness properties, oral bioavailability, solubility in aqueous and lipophilic environments, and distribution profiles of the API *Roseofungin* candidates. 

#### 2.21.2. Toxicological Profile of *Roseofungin*

The prediction method of ProTox3 available online: https://tox.charite.de/protox3/index.php?site=compound_search_similarity (accessed on 20 December 2024), relies on analyzing the similarity between compounds with known median lethal doses (LD_50_) and identifying toxic fragments, offering a novel strategy for toxicity prediction. The ProTox3 methods have been rigorously tested using a diverse external validation set, demonstrating robust performance with the sensitivity, specificity, and precision of 76%, 95%, and 75%, respectively. These results highlight their effectiveness and potential superiority over other toxicity prediction tools, suggesting their applicability to various compound classes [40].

### 2.22. Microbiological Purity

The *Roseofungin* must meet the requirements of the State Pharmacopeia of Kazakhstan 1, V.1, 5.1.4, category 2. A total of 1 g of the drug must not contain more than 100 anaerobic bacteria and fungi (in total), and no more than 10 enterobacteria and some other Gram-negative bacteria is allowed. Moreover, in 1 g of the *Roseofungin*, the presence of *Pseudomonus aeruginosa*, *Staphyloccus aureus* is not allowed.

### 2.23. Acute Toxicity of Roseofungin Determination

To determine acute toxicity, the following experimental groups of animals were formed: 4 groups of animals (5 animals each) were administered different test doses of the *Roseofungin* substance [41]. The animals were monitored for mortality or toxic signs every 2 h on the first day during working hours and every 24 h on the subsequent days. All animals were observed for 14 days following the administration of the test substance solutions. During this observation period, signs of acute toxic effects associated with exposure to the test substance were assessed. Clinical signs of toxicity were recorded as they appeared, including their onset, severity, and duration. These signs included uncoordinated movements, lacrimation, salivation, loose stools, vomiting, piloerection, huddling, prostration, tremors, difficulty breathing, hunched posture, inhibited response to external stimuli, refusal to eat, pale mucous membranes, convulsions, recumbency, and decreased skeletal muscle tone. All deviations in the behaviour and general condition of the animals were documented.

Animal body weight was measured immediately before dosing and then weekly throughout the observation period. Regular monitoring was conducted to prevent deaths due to cannibalism or tissue autolysis. After 14 days of observation, the animals were euthanized, autopsied, and subjected to macroscopic examination. The internal organs, including the liver, lungs, spleen, kidneys, heart, thymus, and adrenal glands, were weighed. Relative weight coefficients were calculated, and the data were statistically analyzed [42].

### 2.24. Test Result Evaluation Criteria

The results of the studies conducted after a single oral administration of *Roseofungin* substance solutions to mice at doses of 50.0 mg/kg, 300.0 mg/kg, 1000.0 mg/kg, and 2000.0 mg/kg of body weight were evaluated based on the following criteria: Animal survival/mortality during the 14-day observation period, body weight of animals receiving the test solutions, somatic parameters, macroscopic assessment of internal organs, and weight coefficients of internal organs.

### 2.25. Analysis of Weight Characteristics

The animals were weighed at the start of the experiment, one week after the administration of the test solutions, and at the conclusion of the experiment on day 15. Body weight was measured in grams. Internal organs, including the liver, lungs, spleen, kidneys, heart, thymus, and adrenal glands, were weighed to calculate the relative mass coefficients of the organs. Statistical analysis of the data was performed.

### 2.26. Analysis of Somatic Parameters

Changes in external somatic signs, such as coat condition, colour of oral mucosa, and other physical indicators, were recorded during daily observations of the animals throughout the 14-day observation period. The parameters observed, any toxic effects detected during the acute toxicity study, and changes in these signs were documented according to SOP-PHT-021. Observations were recorded in primary data sheets.

### 2.27. Macroscopic Examination of Internal Organs

At the end of the study (14 days after the administration of the test solutions) [43], all surviving animals were euthanized in accordance with SOP-PHT-016. Macroscopic examinations were performed in line with SOP-PHT-019. During the macroscopic examination, the condition of the external integuments, physiological openings, regional lymph nodes and their ducts, as well as internal organs and tissues, was assessed. After autopsy, internal organs—including the liver, lungs, spleen, kidneys, heart, thymus, and adrenal glands—were weighed [44].

### 2.28. Interpretation of Test Results

The primary objective of the acute toxicity study is to determine the tolerated, toxic, and lethal doses of the pharmacological substance, as well as the causes of animal deaths. The key parameter of acute toxicity is DL50, defined as the dose of a substance that causes the death of 50% of animals in the observed group.

The toxicity class of the tested substance is determined in accordance with the international classification system GHS (Globally Harmonized System of Classification and Labelling of Chemicals: Acute Toxicity Hazard Categories).

### 2.29. Statistical Analysis of Data

Statistical processing of the results was carried out using the Microsoft Excel 97 programme. Intergroup differences were assessed by the nonparametric Mann–Whitney U-test. In the case of pairwise related groups, the nonparametric Wilcoxon test was applied.

## 3. Results and Discussion

The *Roseofungin* substance was described as an amorphous powder and light-to-dark yellow in colour [45,46], which is well-soluble in DMSO, pyridine, and acetic acid; soluble in ethyl alcohol 96%, butanol, and acetone saturated with water; slightly soluble in chloroform; and practically insoluble in petroleum ether and water [46,47,48]. Its decomposition temperature and specific optical rotation were determined, and some qualitative reactions were examined [46,49].

Physico-chemical parameters of the substance, such as its description, solubility, specific optical rotation, and melting point, were examined more than 30 years ago and therefore necessary to be confirmed or corrected. In addition, the instruments used to determine the specific optical rotation and melting point provided greater accuracy in this study. Solubility was studied by L.A. Vetlugina et al.; however, that study is limited to water and organic solvents that do not cover technological needs (insoluble in water and soluble in fatty oils). In addition, some utilized organic solvents are toxic, and their use in technological processes is impractical and dangerous and environmentally unfriendly. The list of solvents was therefore justifiably expanded for the purpose of new drug development.

**Figure 1:**
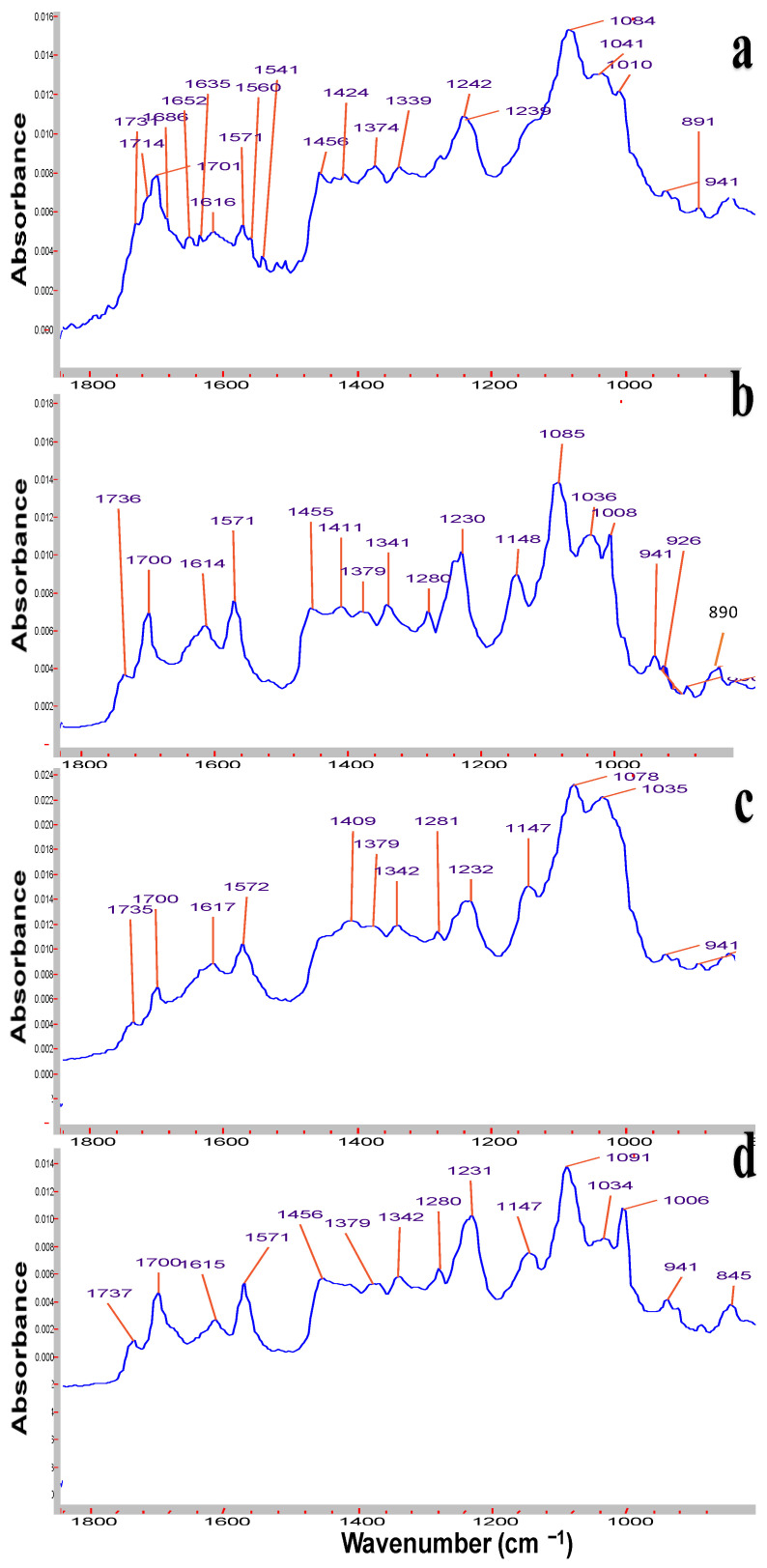
FTIR spectra of *Roseofungin* substance: (**a**) stored for 48 months; (**b**) series N 96 stored for 15 months; (**c**) series N 97 stored for 15 months; and (**d**) series N 102 stored for 14 months.

Symmetrical and asymmetrical frequencies at 1615 and 1570 cm^−1^ correspond to conjugated double bonds C=C (Figure 1). Peaks at 1737 and 1700 cm^−1^ are attributed to carbonyl groups of lactone and the 1700 cm^−1^ signal of keto groups. The C-O stretching mode gives rise to strong absorption bands in the region 1260–1000 cm^−1^ of alcohol groups, and the valent stretching frequency of =C-H group are revealed at 3011–3026 cm^−1^ [45]. From the FTIR spectrum of the relatively old sample of *Roseofungin* (Figure 1a), additional signals appeared at 1685 and 1651 cm^−1^, which most probably related to additional keto groups, the product of oxidation of alcohol groups, and the accumulation of impurity over storage time. A number of signals of alcohol groups overlay each other and are illustrated as a broad peak in the range of 3250–3400 cm^−1^. Previously so-called grey impurity was identified in the *Roseofungin*, which may be related to the product of hydrolysis of the lactone group to the carboxyl group, illustrating the peak at 1635 cm^−1^. Moreover, the sample of *Roseofungin* (Figure 1a) is absent of the signal of the =C-H group at 3011 cm^−1^, which most probably is related to the oxidation process by oxygen and decreases the overall intensity of the signal. A comparative analysis of FTIR spectra of the active pharmaceutical substance at different stages of storage revealed that there is accumulation of degradation products over time, which can be clearly observed using FTIR. One can conclude that three FTIR spectra series of *Roseofungin* 96, 97, and 102 are identical (Figure 1a–d). The frequency of deformational vibration of the hydroxyl group at 1148 cm^−1^ (Figure 1d) disappeared or overplayed with others after a prolonged storage of the substance (Figure 1a). Moreover, the frequency at 1232 cm^−1^ shifted to 1242 cm^−1^, also indicating change in the chemical structure. Physico-chemical and pharmaco-technological parameters of the *Roseofungin* are presented in Table 4.

**Figure 2:**
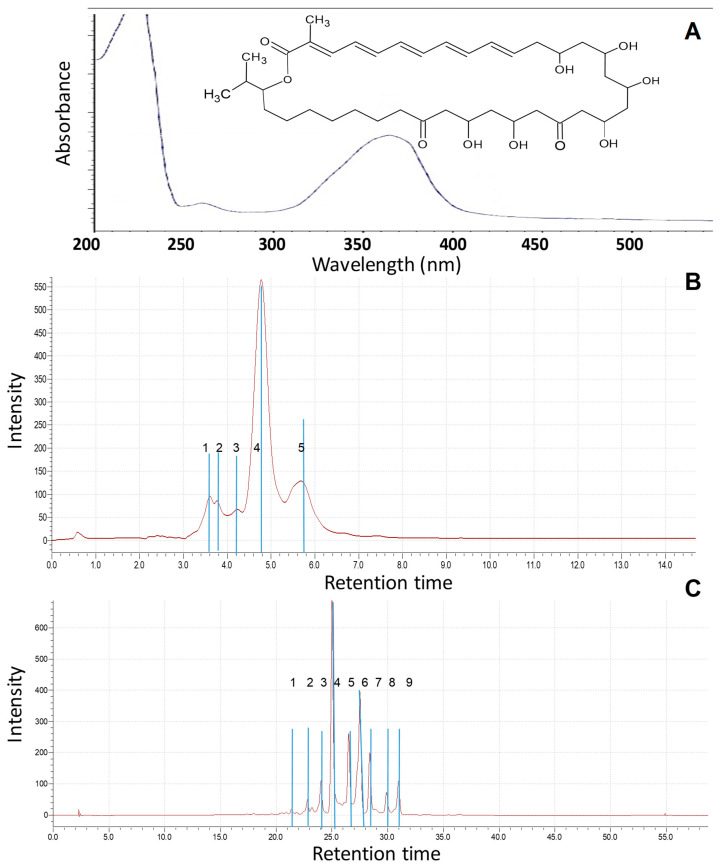
Physico chemical parameters of *Roseofungin*: (**A**) UV spectrum; (**B**) HPLC chromatogram in isocratic mode (acetonitrile water with formic acid 60/40 at flow rate of 0.5 mL/min); (**C**) HPLC chromatogram (acetonitrile TFA water, acetonitrile in gradient mode at flow rate of 1 mL/min).

The absorption maxima at 353 nm for the substance was dissolved in 96% ethyl alcohol and determined in the ultraviolet region (Figure 2A). These data allow us to preliminarily establish the structure of functional groups of the substance and determine its physical properties [45,46,49].

Previously, various mobile phases, including methanol–water, methanol–acetate buffer (0.05 M, pH 4.7), and acetonitrile–acetate buffer, were examined under both isocratic and gradient elution conditions for the analysis of *Roseofungin*. The optimal separation of the complex was achieved using a mobile phase composed of acetonitrile and acetate buffer in a 45:55 ratio, with detection performed at 360 nm [19]. The retention time for the substance in isocratic mode at the eluent composition of 60/40 acetonitrile—water was 3.1 min. In order to estimate quantitatively any impurities (technical, degradation products) in the substance, additional HPLC-MS analysis was carried out at the following elution condition: for 1 h at the composition of 70/30 acetonitrile—water at 30 °C. There were nine main peaks observed within the retention time range of 23–32 min, indicating the composition of the substance (Figure 2B,C).

**Figure 3:**
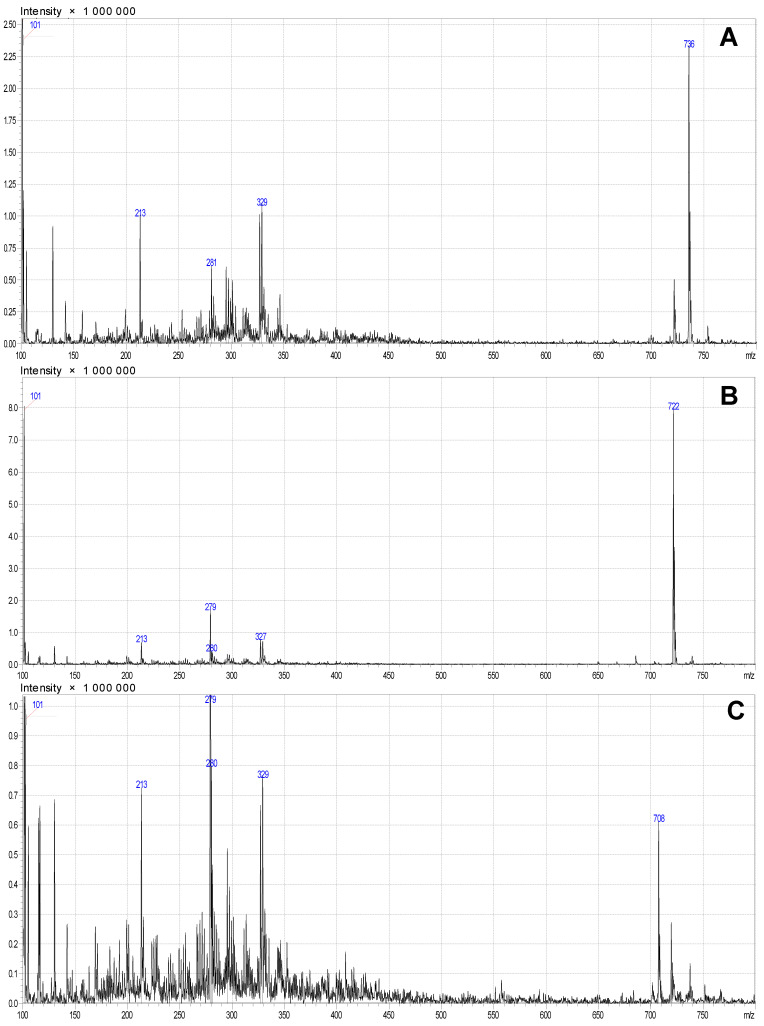
Mass spectrogram of different fractions of *Roseofungin*: (**A**) at retention time 28.4 min, 29.9 min, and 31 min; (**B**) at retention time 24 min, 25 min, 26.5 min, and 27.5 min; and (**C**) mass spectrogram.

The chemical name of *Roseofungin* is 13,15,19,21,23,25-hexahydroxy-2,36-dimethyl-27-oxo-2,4,6,8,10-pentane-17,21-epoxyhepta-triacontane-35-olide (Figure 3A), the empirical formula is C_38_H_68_O_10_, and its molecular weight is 690.9 g/mol [19,20,21]. It appears that there are some variations in of structures.

**Figure 4:**
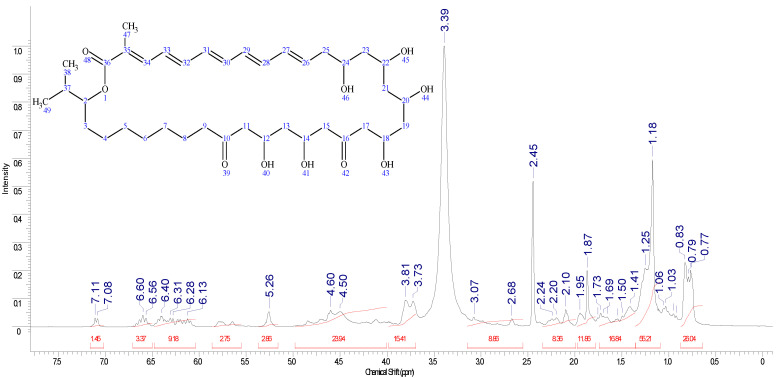
^1^H NMR spectrum of *Roseofungin* in d_6_-DMSO.

General information that can be obtained from ^1^H NMR spectrum characteristic signals of duplets of methyl groups in the range of 0.77–0.82 ppm H-38,38,38 and H-49,49,49 isopropyl fragments of the structure. As expected, H-13,13, H-21,21 and H-23,23 methylen protons have a chemical shift in the field of 1.18–1.25 ppm as multiplet signals, and intensive singlets at 2.45 and 3.39 ppm are related to signals of solvent. Because there are several conformations of the compound, it is impossible to identify each single signal, and it is not necessary, as we use some specific signals and compare integrals between them to confirm the structure. It is unlikely to observe alcohol protons due to their exchange to deuterium. Alcohol groups along with keto and ester groups interact with each other via the formation of hydrogen bonding and supramolecular interactions with methylene groups, and, therefore, there is a high possibility that some of predicted signals appear with a different chemical shift. To facilitate the interpretation of the complicated NMR spectrum, we compare it with the predicted spectrum (Figure 4). Thus, methylene protons H-7,7 and H-6,6 were not observed in the predicted region at 0.24–0.77 ppm that was indicated on the different chemical structure (Figure S1). The chemical structure of *Roseofungin* using mass spectrometry and NMR spectroscopy was studied [19,20,21].

There is a positive correlation between the polarity (or dielectric constant) of a solvent and the solubility of *Roseofungin*, with highly polar solvents being more effective. Exceptions might arise due to specific solvent–solute interactions beyond the dielectric constant or polarity (Table 5). Some solvents with comparable dielectric constants, like PEG 400 (12.5) and pyridine (12.4), exhibit different levels of solubility (VSS for PEG 400 and FS for pyridine). This may relate to other factors like hydrogen bonding, molecular size, or specific interactions between *Roseofungin* and the solvent.

*Roseofungin* represents as a light-to-dark yellow powder with a specific odour, and its melting point is in the range of 104.7 to 110.7 °C (Table 4). *Roseofungin* solubility results in various solvents (Table 5). Based on these results, it was established that the APS *Roseofungin* is freely soluble in DMSO, ethyl alcohol (96%), pyridine, chloroform, dichloromethane, acetic acid, and sunflower oil; soluble (S) in butanol and benzene; slightly soluble in n-hexane; and very slightly soluble in water.

Hydrophilic and lipophilic bases were examined for further pharmaceutical development of new dosage forms. It was found that *Roseofungin* is freely soluble in lipophilic bases (all Witepsol brands, cocoa butter, solid confectionery fat) at a temperature of 35 ± 1 °C and very slightly soluble in hydrophilic polymers such as PEG.

The solubility of the original *Roseofungin* in various solvents and bases used to manufacture ointment was determined (Table 5), and, as expected, the substance revealed pronounced lipophilic properties. It should be noted that the substance is partially wettable by water. The aforementioned excipients for the proper development of new dosage forms according to the solubility were taken into account.

The specific optical rotation measured [45] in 1977 is comparable to the findings of this study; the deviations are approximately 1°/mL/g^−1^/dm^−1^. The values for the specific optical rotations of the substance (Table 4) compared with the literature data were as follows: ${\left[a\right]}_{589}^{23}$ −52 [36], ${\left[a\right]}_{589}^{20}$ −53.088 (0.25% solution in pyridine); ${\left[a\right]}_{589}^{23}$ −68 [36], ${\left[a\right]}_{589}^{20}$ −68.99 (0.25% solution in methanol); and ${\left[a\right]}_{589}^{23}$ −27 [36], ${\left[a\right]}_{589}^{20}$ −28.361 (1% solution in dioxane).

**Figure 5:**
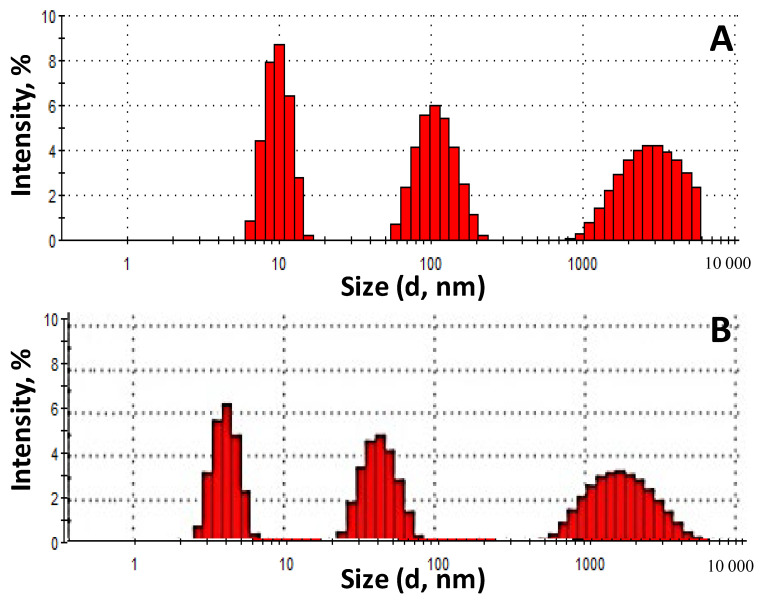
Fractional composition diagrams for the *Roseofungin* in water ((**A**)—before grinding, (**B**)—after grinding).

The fractional composition of the *Roseofungin* was determined before grinding (a) and after grinding for 60 s (b) using laser diffraction (Figure 5). In accordance with the pharmacopeia article [35], two types of *Roseofungin* powder can be classified as the finest because the size of the largest particles does not exceed 10 μm. The fractional composition before grinding is represented by the following particle distribution: a fraction in the range of 900–6000 nm—36.4%, a fraction in the range of 50–200 nm—32.3%, and a fraction in the range of 6–20 nm—31.3%. Meanwhile, after grinding: a fraction in the range of 500–6000 nm increased to 41.3%, a fraction in the range of 20–80 nm—33.6%, and the fraction 3–6 nm was occupied a quarter. The fractional composition has a wide range in both cases, which indicates its heterogeneity. To obtain a homogeneous nanodispersed powder, it is necessary to extend the length of the grinding time, but this entails a rise in technological losses and an increase in the electrostatic voltage between molecules. Thus, this comparative study was carried out to determine the expediency of grinding using a centrifuge. The results of the analysis indicated the possibility of not including the grinding operation in the technological process during substance production due to the optimal particle size and the reduction in losses in the process flow. The possibility of dissolving the substance in approved solvents and introducing the concentrate into the dosage form is therefore the most appropriate.

**Figure 6:**
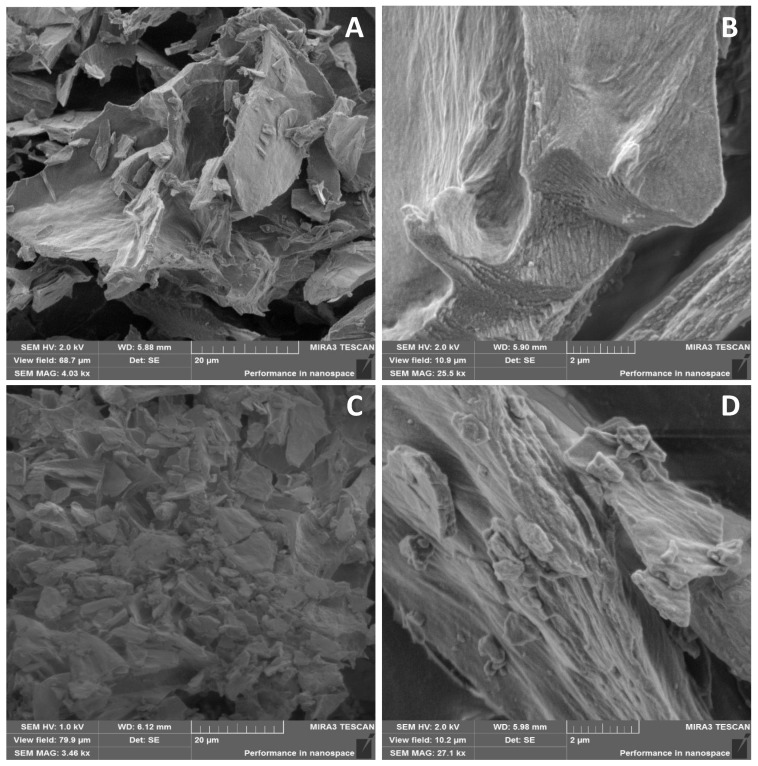
SEM images of the *Roseofungin*: (**A**)—untreated substance (20 µm), (**B**)—untreated substance (2 µm), and (**C**)—grinded substance (20 µm), (**D**)—grinded substance (2 µm).

The *Roseofungin* particle shape is shown in the SEM image in Figure 6. The substance has a predominantly lamellar structure (Figure 6A), which can be seen upon magnification of ×500 times (Figure 6B). After grinding the substance, plates with finer dispersions were obtained (Figure 6C). As seen in the SEM image at higher magnification, the surface of the plate becomes scabrous after grinding (Figure 6D). The shape and size of substance particles can also affect the pharmacological and technological properties of powders [36,37]. Crystallographic data of the drug substance particles obtained during this study of their complex surface showed that there is considerable interparticle friction, which creates electrostatic tension between molecules and the additional adhesion of particles. This circumstance significantly affects the fluidity/flowability of the substance. This study revealed the absence of interparticle friction at a substance moisture content of 1.41%. Electrostatic voltage also adversely affects the technological process itself when loading the drug substance directly into the reactor through the charging hopper, which leads to significant material loss. In this regard, before the direct introduction of the substance into the base, it is necessary that the substance be either dissolved in a regulated solvent or in part of the base; only after this step, can it be loaded into the reactor with the base.

The evaluation of the angle of repose was impossible on the PTG S4 automated powder and granule flow analyser (PharmaTest, Hainburg, Germany) due to the lack of flowability. The very poor flowability of the powder was confirmed by the calculated Hausner ratio (V = 1.42, *p* = 1.42) and Carr compressibility index (V = 41.64, *p* = 41.67).

**Figure 7:**
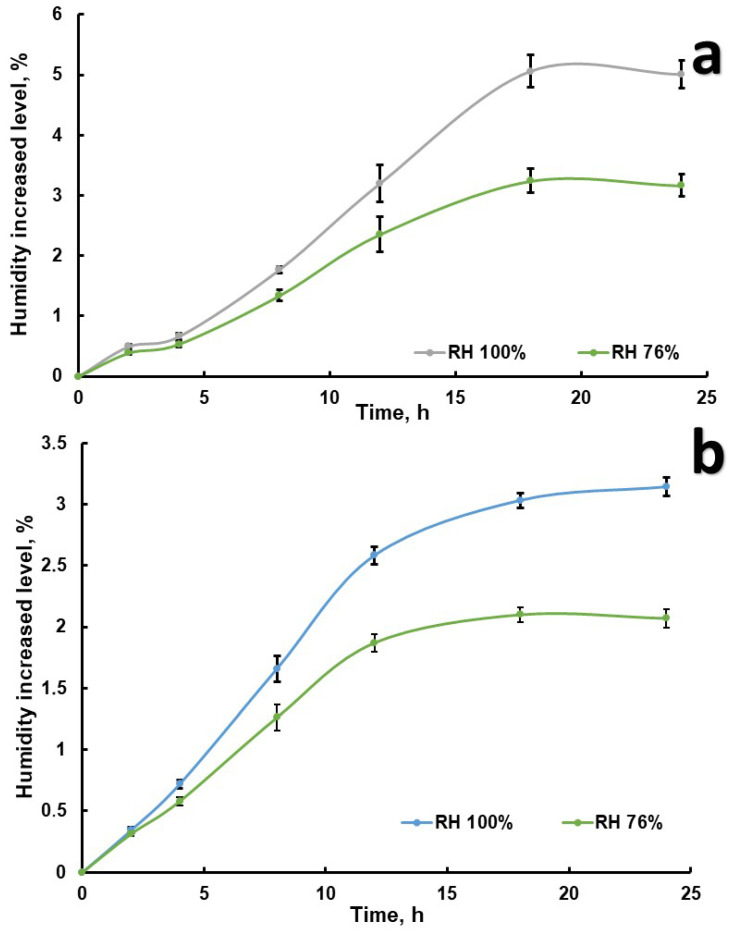
Hygroscopicity diagrams for *Roseofungin* ((**a**)—determination of moisture content using an electronic moisture analyser, (**b**)—mass measurement method).

The results correlated with each other, indicating that the substance was hygroscopic (Figure 7). The end of the experiment reached equilibrium of the moisture absorption within 18–20 h, and the estimated standardized mean difference between the subgroups was in the range from 0.95% to 2.28%. In this manuscript, we do not discuss optimization of the granulation techniques; rather, we state that, at the moment on the production line, the followability of the substance has some parameters. There was comprehensive investigation in order to obtain the appropriate dispersion degree and density for the technological process; unfortunately, that part of the recrystallization process is a company secret. There are no technological issues with processing of the substance due to good solubility in DMSO and following processing to ointment.

**Figure 8:**
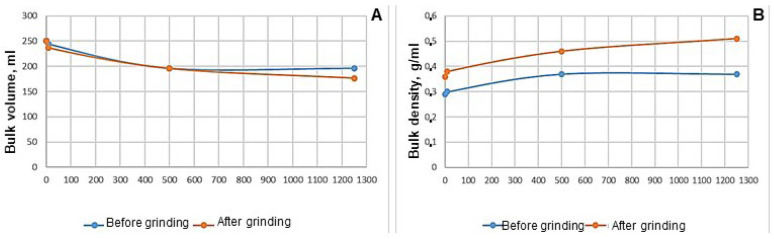
Diagrams for the *Roseofungin* before and after grinding: (**A**)—bulk volume and (**B**)—bulk density.

The relative moisture content was 1.63 ± 0.06% with a regulated level of no more than 2.5%. In total, 74.4 g of the substance was taken before grinding, and 90 g of the substance was taken after grinding, which required a volume (V_0_) of 250 mL (Figure 8).

The bulk volume values of *Roseofungin* (Figure 8) show insignificant differences between the values obtained after 0 and 500 taps, with values ranging from 0 to 0.7 units. Significant differences in bulk volume were noted after 10 and 1250 taps, and the values ranged from 7.2 to 23.3 units. This indicates a greater degree of shrinkage of the finely divided substances. With untreated and grinded substances, the following dependence remained: when the bulk volume decreases, the bulk density increases. It was established that the bulk density of the powder with a lower degree of dispersion was 1.2 times higher than the one with a higher degree of dispersion. *Roseofungin* types have the ability to settle and are considered light powders.

The true density of *Roseofungin* was 1.1938 + 0.01 g/cm^3^, the relative density was 30.16 + 0.1%, and the porosity reached 57.28 + 0.02%. The relative density characterizes the fraction of the space occupied by the powder material. As its value was in the range of 12 to 40%, the powder has anisodiametric particles that are stacked relatively loosely. The porosity of the system increases with loose stacking. Voids (pores) in the substance occupy more than 50% of the volume. The following pattern was observed: the lower the tapped density, the greater the porosity of the mass, and the greater its volume. The results of this study correlated with the data on nystatin and levorin.

As mentioned earlier, this substance is currently registered in the territory of the Republic of Kazakhstan, and its quality is regulated by the requirements of the analytical normative document No. 42-8181-17, which was drawn up in accordance with the requirements [37,38]; it was approved by the regulatory authorities. The product should be stored at 2 °C to 8 °C, and the shelf life of the finished product is 3 years.

### Biological Activity Evaluation of Roseofungin

*Roseofungin* exhibits antifungal activity against various pathogens responsible for both superficial and deep mycoses, including trichophytosis, microsporia, favus, candidiasis, cryptococcosis, sporotrichosis, chromomycosis, and aspergillosis, with effective concentrations ranging from 0.5 to 12.5 mg/L [21,22]. Its activity against *Candida albicans* and non-albicans species falls within the range of 1.11–1.65 mg/L. Additionally, *Roseofungin* demonstrates efficacy against Aspergillus at concentrations of 4.6–8.33 mg/mL, surpassing the effectiveness of widely available commercial antifungal drugs [23]. The median MICs of nystatin for the tested isolates ranged from 4 to 8 μg/mL. Specifically, the median MIC for *Candida albicans* 2733A and *Candida krusei* 37-5696A was 8 μg/mL, while all other isolates exhibited an MIC of 4 μg/mL [50]. For amphotericin B, the M27-P method produced a consistently narrow MIC range of 0.125 to 1 μg/mL, with no significant variations in susceptibility among different species. In 90% of isolates, the MIC values obtained through broth microdilution differed by no more than one dilution from those determined using the M27-P method [51]. Among organisms treated with *natamycin*, *Aspergillus flavus* exhibited the highest MIC_50_ and MIC_90_ values, measuring 32 μg/mL (95% CI: 32–64 mg/L) and 64 mg/L (95% CI: 32–64 mg/L), respectively. In the case of *voriconazole* treatment, *Fusarium* species had the highest MIC_50_ of 4 μg/mL (95% CI: 2–4 μg/mL) and MIC_90_ of 8 mg/L (95% CI: 8–16 mg/L) [52].

Based on the acute toxicity studies conducted with a single oral administration of *Roseofungin* at doses of 50 mg/kg; 300 mg/kg; 1000 mg/kg; and 2000 mg/kg of body weight, the following outcome can be drawn: (a) the death of animals was not noted at all doses used, which does not allow calculating the estimated average lethal dose of LD_50_; and (b) toxicity class can be defined as 5-non-toxic substance, since, at a maximum dose of 2000 mg/kg, there was no death of any animal from the group. According to ProTox3 software https://tox.charite.de/protox3/index.php?site=compound_search_similarity (accessed on 20 December 2024), in silico predicted at oral administration LD_50_ was found at 6060 mg/kg [53].

Macroscopic Observations Following a Single Oral Administration of *Roseofungin*.

A macroscopic examination of experimental animals and administered single doses of *Roseofungin* solutions at 50 mg/kg, 300 mg/kg, 1000 mg/kg, and 2000 mg/kg revealed no abnormalities in the internal organs. An external assessment of body build, fur palpation, inspection of the skin, and mucous membranes (oral cavity, eyes, conjunctiva, external genitalia, and anus) revealed no visible abnormalities (Figure S2).

Findings after skin separation axillary and inguinal lymph nodes were not enlarged in any group. The prostate appeared normal in colour and size. The submandibular salivary gland and superficial cervical lymph nodes were differentiated, without observable changes. The parotid salivary glands and cranioventral lymph nodes remained unchanged.

Findings after abdominal wall opening: The topographic arrangement of thoracic, abdominal, and pelvic organs was typical, with no detected pathologies. The thymus was located above the heart, at the base of the neck; it was soft and greyish-white.

The thyroid gland area has no visible changes or exudate observed. The heart had a normal appearance, the left ventricle was not enlarged on the cross-section, and the pericardium contained no fluid. The lungs and pleura were smooth but slightly dry. The lungs (right and left) were collapsed, unevenly coloured, and free of seals or air bubbles. The aorta was clearly visualized, with open and unobstructed cavities. The pancreas appeared as yellowish in colour, visualized as a network within the greater omentum near the small intestine. Necrosis or abnormal formations were not observed. In the stomach, upon incision along the greater curvature and washing with physiological solution, typical folds were observed without hyperemia or ulcers. The small and large intestines were unchanged and contained chyme. The mesenteric lymph nodes were normal. The spleen was typically located, oblong in shape, and dark cherry in colour with a moderately dense consistency. Its capsule was thin, tightly adherent, and did not leave marks upon pressure or scrapings when cross-sectioned. The white pulp follicles were visible, and no ulcers, hemorrhages, or pathological changes were present. Adhesions to the stomach, diaphragm, or other organs were not noted. The liver surface was smooth and uniformly coloured, with a soft, elastic consistency. The gallbladder was olive-coloured and contained bile. Each liver lobe exhibited normal histoarchitecture, moderately full-blooded parenchyma, and no scrapings on the scalpel during cross-sectioning. The kidney surfaces were brownish, shiny, and smooth, with a thin, transparent capsule easily removed. The kidneys were normal in size and appearance, with clearly distinguishable darker outer cortices and lighter inner medullae on longitudinal sections. Renal pelvises were free and not dilated. The adrenal glands were visualized and approximately the size of millet grains. In some animals, the bladder contained light urine. The scrotal organ complex was typical. Ovaries were clearly visible, uterine horns were patent, and the vaginal cavity was free. After the evaluation of *Roseofungin* safety, it is interesting to estimate other possible biological effects, as well as absorption, distribution, metabolism, and excretion. Histological studies were not conducted, as no visible pathological changes in the internal organs were observed during macroscopic examination.

Sub-chronic or chronic toxicity studies were not performed to assess potential cumulative toxicological risks, as the drug formulation is for external use only. Of course, these experiments are interesting to perform, and that information will be sufficient for another non-clinical investigation paper. Histopathological studies are important, and these will be studied in future. However, before an in vivo study, we have to provide in silico evidence of safety of the substance.

**Figure 9:**
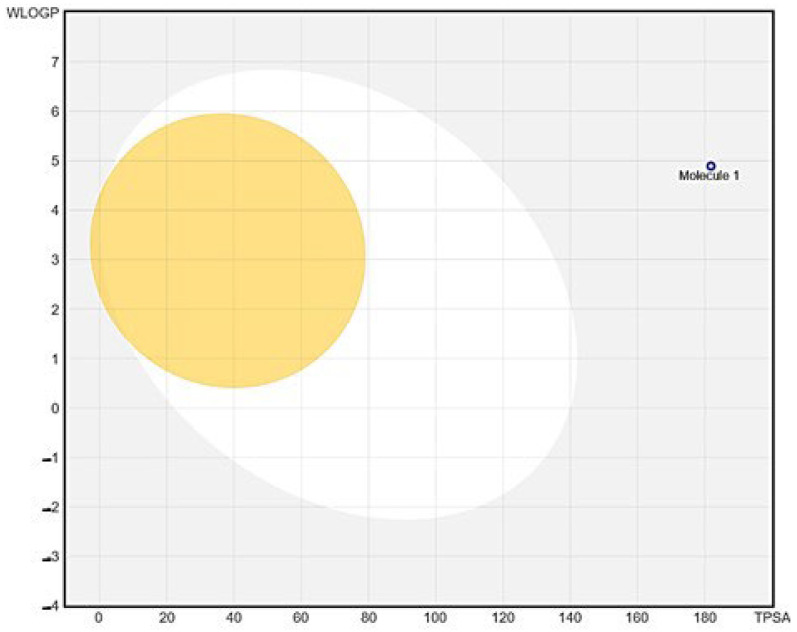
BOILED-Egg illustration for predicting the absorption of compounds in the gastrointestinal tract (white area) and penetration into the brain (yellow area) in silico (http://www.swissadme.ch, accessed on 20 December 2024).

The absorption blood–brain barrier (BBB) of *Roseofungin* is predicted as BBB- (not likely to cross the blood–brain barrier), with a probability of 0.53 (Table 6, Figure 9). This suggests minimal central nervous system penetration. Human Intestinal Absorption (HIA) classified as HIA+ (likely absorbed in the intestine), with a probability of 0.59, indicates potential bioavailability upon oral administration. Caco-2 permeability (low permeability across intestinal epithelium) has a probability of 0.51. This may imply limited absorption efficiency. P-glycoprotein Substrate/Inhibitor *Roseofungin* is a substrate (0.685), but non-inhibitor probabilities 0.89 and 0.81, respectively, suggest potential efflux by P-glycoprotein without significant interference with its function. Distribution is predicted as subcellular localization, predominantly in mitochondria (0.72), which could influence its mechanism of action or toxicity profile in mitochondrial processes. Regarding metabolism, we can state following: (a) *Roseofungin* is not a substrate for CYP450 2C9 (0.81) and CYP450 2D6 (0.88), but it is a substrate for CYP450 3A4 (0.61); (b) it is not an inhibitor for CYP450 1A2, 2C9, 2D6, 2C19, or 3A4 (non-inhibitor probabilities ranging from 0.613 to 0.916). *Roseofungin* shows low CYP inhibitory promiscuity (0.964), indicating the minimal risk of broad CYP450-related drug interactions. Toxicity by ADMET assessment was recently utilized for the selection of lead substance having anti-acne activity against *Propionium acnes GehA* lipase bacteria. Bo Zhang et al.’s application of a computational approach with filtering potential compounds using admetSAR 118 bioactive compounds from 11 herbs was analyzed [54]. Previously, we evaluated acute toxicity and their ADMET profiles for 25 hydrazide derivatives of N-piperidyl propanoic acid and N-morpholyl propanoic acids in silico [55]. It is expected that related excretion properties are readily biodegradable (0.79), suggesting it may not persist significantly in the environment post-excretion, most probably due to double bonds and an easily hydrolysable ester group. Pharmacokinetic properties were studied using the BOILED-Egg model, which allows estimating passive gastrointestinal absorption and brain penetration by calculating their lipophilicity and polarity described by the n-octanol/water partition coefficient (log P) and the polar surface area. The white area corresponds to a high probability of passive absorption in the gastrointestinal tract, and the yellow area corresponds to a high probability of brain penetration [55].

In silico toxicity evaluation was illustrated as a weak inhibitor (0.906) or non-inhibitor (0.908), and Human Ether-a-go-go-Related Gene (hERG) inhibition indicated low cardiotoxic potential related to hERG channel blocking. Non-AMES toxic (0.866) and non-carcinogenic (0.92) suggested the lack of mutagenic or carcinogenic properties. Even though *Roseofungin* was proved to be safe to humans, it is necessary to check the environmental impact. Toxicity software (ProTox3) predicted high toxicity to fish (0.764), Tetrahymena Pyriformis (0.93), and honeybees (0.785); therefore, any wastewater at the pharmaceutical industry must be properly purified because of rising environmental concerns. Acute oral toxicity was attributed to category 3 with a probability of 0.44, indicating moderate oral toxicity. *Roseofungin* classified as non-required (0.71), supporting its non-carcinogenic status (Table 6). Caco-2 permeability LogPapp 0.6006 cm/s indicates moderate intestinal permeability. A higher value would suggest better absorption through the intestinal lining. Rat acute toxicity is LD50 2.6839 mol/kg) while fish toxicity is pLC50 1.712 mg/L. Lower pLC50 values indicate higher toxicity to fish populations (Table 7).

**Figure 10:**
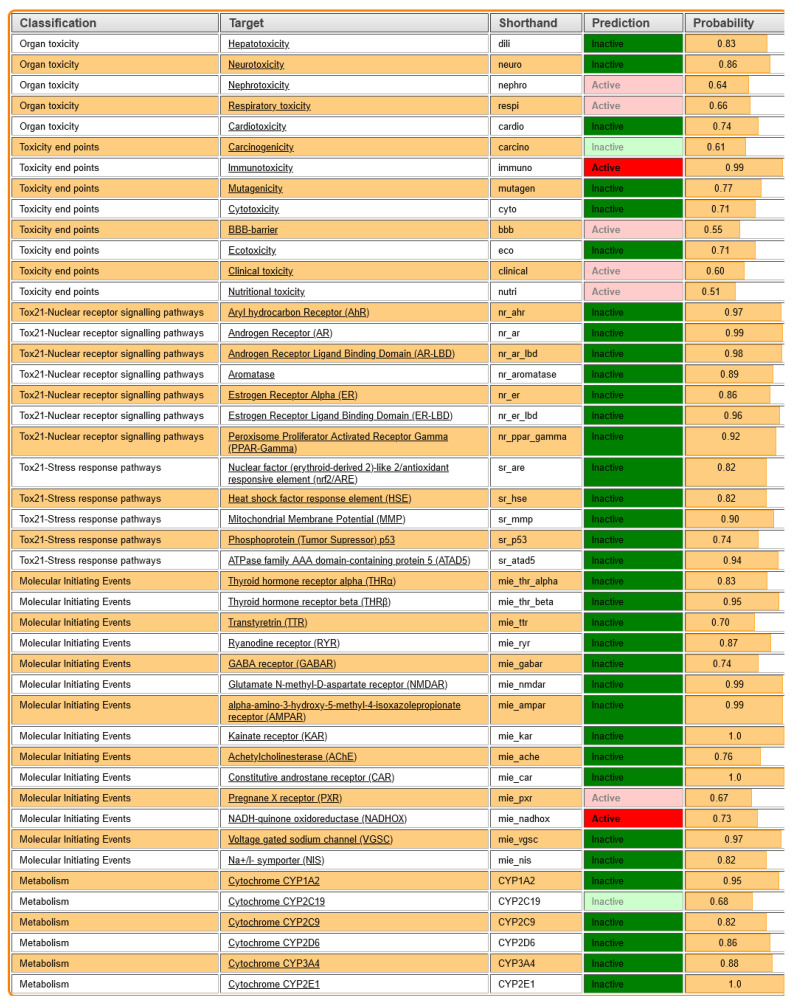
Toxicological profile of *Roseofungin* possible toxicity targets, which is based on an in-house collection of protein–ligand-based pharmacophore models (‘toxicophores’) for targets associated with adverse drug reactions (ProTox3 software).

Figure 10 summarizes the toxicity predictions for *Roseofungin* based on different classifications and targets, providing insight into its potential adverse effects. Hepatotoxicity (dili) was predicted as inactive with a probability of 0.83, indicating a low risk of liver toxicity, that correlates well with the in vivo toxicity study. Neurotoxicity (neuro) was predicted as inactive with a high probability of 0.86, suggesting minimal risk to the nervous system. Nephrotoxicity (nephro) was foreseen as active with a relatively low probability of 0.64, indicating a moderate potential for kidney toxicity. The absence of adverse effects was also confirmed by in vivo data Respiratory Toxicity (respi) in silico, expected as active with a probability of 0.66, reflecting a possible respiratory risk; however, it was not confirmed by the in vivo study. Cardiotoxicity (cardio) was foreseen as inactive with a probability of 0.74, suggesting a low likelihood of heart toxicity, which is in agreement with in vivo. Carcinogenicity (carcino) was predicted as inactive with a probability of 0.61, indicating a low cancer risk. To evaluate the in vivo carcinogenicity effect, a longer period of time for observation is required, which was outside of the scope of the current study. From the other side, immunotoxicity (immuno) was predicted as active with a high probability of 0.99, highlighting a significant potential for immune system toxicity. Mutagenicity (mutagen) was predicted as inactive with a probability of 0.77, suggesting minimal risk of causing genetic mutations. Cytotoxicity (cyto) was predicted as inactive with a probability coefficient of 0.71, indicating low toxicity to cells. The BBB was predicted as active with a probability of 0.55, which correlates with the BBB data obtained using other software, showing moderate potential for crossing the blood–brain barrier, which may have implications for neurotoxicity. At the moment, we consider the potential pharmaceutical form as vaginal suppositories and ointment for topical application. 

Clinical Toxicity (clinical) was predicted as active with a probability of 0.60, suggesting a moderate risk of clinical adverse effects. Nutritional Toxicity (nutri) was predicted as active with a lower probability of 0.51, indicating a potential, though minimal, impact on nutritional health (Table 8).

Based on the results of this acute toxicity study of the *Roseofungin* substance in vivo, administered orally at the doses specified in the study plan (50 mg/kg, 300.0 mg/kg, 1000 mg/kg, and 2000 mg/kg of body weight), the following summary can be drawn: over the 2-week observation period, no biological reactivity or animal deaths were observed following administration of the test substance at any dose. Somatic parameters remained unchanged. This study of body weight dynamics in mice revealed no statistically significant differences between the experimental groups. There were no statistically significant differences in the absolute weights or relative weight coefficients of internal organs between the experimental groups. Macroscopic examination of the mice showed that the location, appearance, and other parameters of the internal organs corresponded to species-specific characteristics (Tables S1–S3). No pathological changes were detected in the internal organs of animals from the experimental groups. The following conclusions can be drawn regarding the acute toxicity of *Roseofungin* with single-dose oral administration at 50 mg/kg, 300 mg/kg, 1000 mg/kg, and 2000 mg/kg. No animal deaths were observed at any of the tested doses, making it impossible to calculate the expected median lethal dose (LD_50_). The toxicity classification for *Roseofungin* has category 5 (non-toxic substance), as no animal deaths occurred even at the maximum dose of 2000 mg/kg.

**Figure 11:**
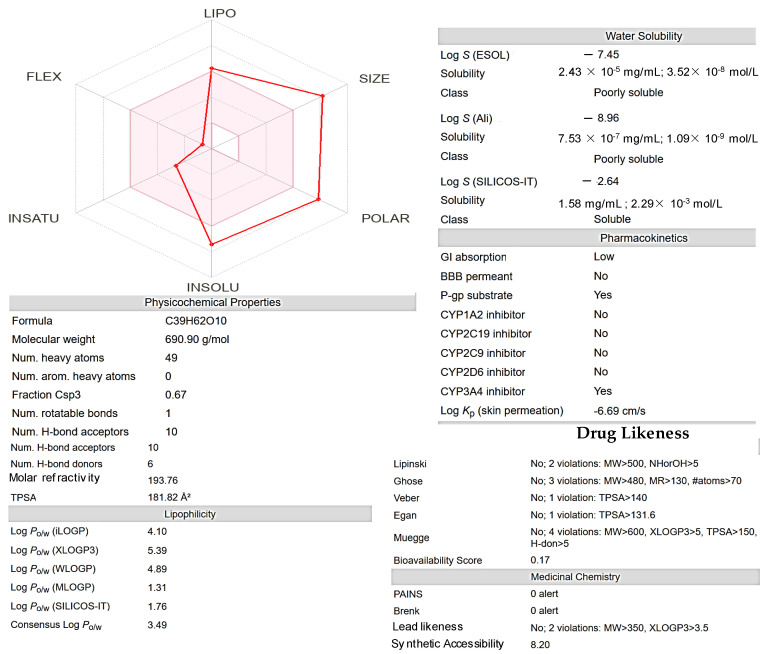
Bioavailability radar and essential physicochemical properties of *Roseofungin* using the SwissADME web resource http://www.swissadme.ch/, accessed on 20 December 2024.

This radar plot visualizes the ADME-related properties of a *Roseofungin* (Figure 11), categorizing its attributes into five key dimensions. LIPO (lipophilicity) measures the compound’s lipophilicity, indicating its ability to dissolve in fats and oils (Log P 4.1, although XLogP3-AA 5.4). A higher value suggests better lipophilicity, which can affect membrane permeability and distribution. SIZE (molecular weight of 690.43 g/mol) and larger values may indicate a bulkier molecule, which could limit absorption or bioavailability. POLAR reflects the polarity of the compound, often associated with the topological polar surface area (TPSA 182Å²). Higher polarity is generally beneficial for solubility in aqueous environments but may hinder membrane permeability. INSOLU (insolubility) and a higher score on this axis may suggest poor solubility in aqueous media, which can reduce bioavailability. INSATU (unsaturation) attributed to the degree of unsaturation (double bonds (*n* = 5)) in the *Roseofungin*. This can affect reactivity and drug likeness. FLEX (flexibility) measures the *Roseofungin*’s molecular flexibility, which can influence binding to biological targets and overall pharmacokinetic behaviour.

**Figure 12:**
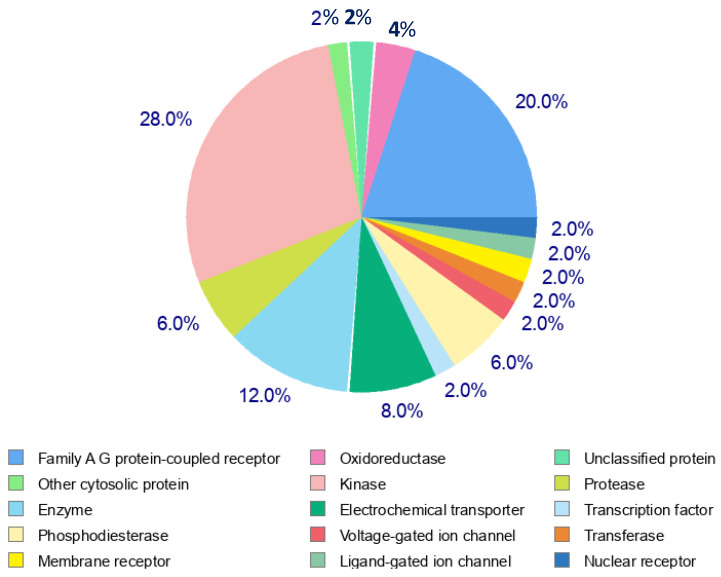
SwissADME target prediction for Roseofungin for homo sapience.

The distribution of target protein classes in the pie chart reveals the following key insights (Figure 12). Family A G Protein-Coupled Receptors represent the largest proportion (28%), highlighting their significant role as drug targets. Enzymes (20%) and kinases (12%) collectively contribute to a substantial portion of the targets, emphasizing their importance in biochemical processes and therapeutic interventions. Membrane receptors (8%) and phosphodiesterases (6%) further demonstrate the relevance of cellular signalling and regulation in the target profile. Smaller contributions come from oxidoreductases, ligand-gated ion channels, voltage-gated ion channels, electrochemical transporters, and nuclear receptors (each at 2–4%), reflecting diverse target classes with specialized roles. Other cytosolic proteins and unclassified proteins comprise a small percentage, indicating the ongoing exploration of novel and less-characterized targets.

The maximum tolerated dose of orally administered *Roseofungin* was 400 mg/kg. The LD_50_ values for mice after intraperitoneal and intravenous administration were determined to be 140 and 16 mg/kg, respectively [56]. According to the LD_50_ level after intraperitoneal administration, *Roseofungin* is significantly less toxic than other polyenes (examples of drugs). *Roseofungin* is 6–8 times less toxic than *nystatin* (LD_50_ 20–25 mg/kg), levorin (LD_50_: 7–35 mg/kg), *mycoticin* (LD_50_ 10–25 mg/kg), *flavofungin* (LD_50_ 25 mg/kg), etc. [48,57].

The repeated daily intraperitoneal administration of *Roseofungin* to mice at the dose of 5 mg/kg of body weight was well tolerated during the observation period of 10 days, and the daily oral administration of *Roseofungin* at the doses of 50 and 100 mg/kg for 18 days to guinea pigs and at the doses of 50, 150, and 450 mg/kg to rabbits did not cause deterioration in the conditions of the animals or a decrease in their activity [19]. Roseofungin at concentrations of 1, 3, and 5% did not have local irritating effects on the mucous membranes of the eye and intact skin of animals. Moreover, *Roseofungin* did not possess allergenic activity or affect phagocytosis and had mitostatic and lymphotoxic effects [15].

## 4. Conclusions

The pharmaco-technological parameters of the APS Roseofungin were studied. Analysis of the obtained data revealed that the substance has electrostatic properties and does not possess flowability. It is hygroscopic and lipophilic. The APS represents as a light powdery substance with a porosity greater than 50% and possesses the ability to settle. According to in silico modelling and Lipinski rules, the *Roseofungin* exhibits several violations of drug likeness and bioavailability rules, suggesting challenges in oral administration and passive permeability. It has moderate lipophilicity, but its high TPSA and molecular weight may hinder absorption and distribution. We know that Lipinski’s rule states that, in general, an orally active drug has no more than one violation of the following criteria such as the following: no more than 5 hydrogen bond donors (the total number of nitrogen–hydrogen and oxygen–hydrogen bonds); no more than 10 hydrogen bond acceptors (all nitrogen or oxygen atoms); a molecular mass less than 500 Daltons; and a calculated octanol–water partition coefficient (Clog P) that does not exceed 5 [57].

Lipinski formulated a rule based on his observation that most drugs administered orally are relatively small and moderately lipophilic molecules. This rule serves as a guideline to determine whether a molecule with specific pharmacological or biological activity possesses properties that would make it a viable orally active drug in humans. Taking into account the abovementioned, it was proposed that this formulation for topical application should be used only as ointment, due to instability of the structure in gastric fluids as well as poor solubility in water [58].

Additionally, *Roseofungin* shows no structural liabilities (PAINS or Brenk alerts), although synthetic accessibility appears complex. The compound shows favourable absorption and metabolism profiles for human use but limited permeability and central nervous system penetration. It presents low risks for AMES toxicity, carcinogenicity, and CYP450-mediated drug interactions, making it potentially safe for therapeutic applications under controlled usage. In the future, these activities will be tested in vitro on various cell lines and then on animal models. The experimental data enable further work on the proper pharmaceutical development of new drugs containing the original APS *Roseofungin*.

The macroscopic evaluation of experimental animals following a single oral administration of *Roseofungin* solutions at doses of 50.0 mg/kg, 300.0 mg/kg, 1000.0 mg/kg, and 2000.0 mg/kg demonstrated no pathological abnormalities in the examined internal organs or external characteristics. Normal body build, fur condition, skin, and mucous membrane appearance with no visible changes were observed. Unaltered lymph nodes, salivary glands, and topographic arrangement of internal organs were observed across all experimental groups. There were the typical macroscopic features of vital organs, including the heart, lungs, liver, spleen, kidneys, pancreas, and intestines, without any evidence of pathological changes such as hyperemia, necrosis, ulcers, or adhesions. Reproductive and urinary systems appeared normal, with no anomalies in the scrotum, ovaries, uterine horns, or bladder. These findings confirm that *Roseofungin*, even at the maximum dose of 2000 mg/kg, does not induce detectable macroscopic toxic effects in internal or external organs of the tested animals, supporting its classification as a non-toxic substance under the tested conditions. Overall, the data reflect a broad and balanced distribution of target proteins, with a notable emphasis on receptors and enzymes, aligning with their crucial roles in therapeutic applications and biological regulation. Currently, this substance is considered to be utilized in pharmaceutical forms such as vaginal suppositories and ointment for topical application.

## Figures and Tables

**Table 1:** HPLC gradient eluent composition for *Roseofungin* separation.

Time, min	Water (1% TFA), %	ACN (1%TFA), %
0	85	15
5	70	30
30	50	50
50	15	85
53	0	100
55	85	15
60	85	15

**Table 2:** Flowability scale [35,36,37].

Carr Compressibility Index, %	Hausner Ratio	Flowability Characteristic
1–10	1.00–1.11	Very good
11–15	1.12–1.18	Good
16–20	1.19–1.25	Satisfactory
21–25	1.26–1.34	Unsatisfactory
26–31	1.35–1.45	Poor
32–37	1.4–1.59	Very poor
>38	>1.60	Practically non-existent

**Table 3:** Hygroscopicity scale [36].

Moisture (Mass Increase), %	Hygroscopicity Characteristic
Fluid formation is observed due to absorbing enough water	Diffluent
15–more	Very hygroscopic
2–15	Hygroscopic
0.2–2	Low hygroscopicity

**Table 4:** Physico-chemical and pharmaco-technological parameters of *Roseofungin*.

SN	Control Parameters	Results
**Physico-chemical parameters**		
1.	Colour	Light-to-dark yellow
2.	Odour	Specific
3.	Particle size	Lamellar structure
4.	Electrostatic properties	Has electrostatic properties
5.	Specific optical rotation	−53.09 ± 0.53 (0.25% in pyridine), −68.99 ± 0.57 (0.25% in methanol), −28.36 (1% in dioxane)
6.	Melting point, °C	Starting melting point—105 ± 0.46, final melting point—110 ± 0.32
7.	Wettability	Partial
8.	Hygroscopicity	Hygroscopic
**Pharmaco-technological parameters**		
9.	Fractional composition, %	Before grinding: from 900 to 6000 nm—36.4%, from 50 to 200 nm—32.3%, from 6 to 20 nm—31.3% (finest powder) After grinding: from 500 to 6000 nm—41.3%, from 20 to 80 nm—33.6%, from 3 to 6 nm—25.1% (finest powder)
10.	Bulk volume, cm^3^	V_0_ = 250.0, V_10_ = 236.8, V_500_ = 195.7, V_1250_ = 176.5 ± 0.74
11.	Bulk density, g/cm^3^	P_0_ = 0.36, P_10_ = 0.38, P_500_ = 0.46, P_1250_ = 0.51 ± 0.01
12.	Hausner ratio	by V = 1.42, by P = 1.42
13.	Carr compressibility index	by V = 41.64%, by P = 41.67%
14.	Flowability, g/s	∞
15.	Angle of repose, °	Absent
16.	Loss in mass on drying, %	Sample 1—1.44%, Sample 2—1.56%, Sample 3—1.22% Mean value—1.41 ± 0.17%
17.	True density	1.1938 g/cm^3^
18.	Relative density	30.16%
19.	Porosity	57.28%

**Table 5:** Solubility of Roseofungin in different polarity of solvents, FS: freely soluble; S: soluble; SS: slightly soluble; VSS: very slightly soluble; the sign “-” means that conducting of testing in some cases/conditions is inappropriate.

Solvent Name			Temperature Mode, °C		
	*Polarity of Solvent*, Dielectric Point	Dipole Moment	20 ± 1	35 ± 1	60 ± 1
DMSO	46.7	3.96	FS	-	-
Tween 80	8.75 [41]		FS	-	-
Glycerine	42.5		-	FS	-
Lanoline	*4.2*		-	FS	-
PEG 400	*12.5* [42]	-	VSS	-	-
PEG 1500	-	-	-	-	VSS
Acetic acid	6.2	1.74	FS	-	-
Ethyl alcohol (96%)	*24.3*	*1.69 D*	FS	-	-
Purified water	80	1.85	VSS	-	-
Sunflower oil			FS	-	-
Cocoa butter			-	FS	-
Solid fat			-	FS	-
Witepsol H, W, S, E			-	FS	-
Benzene	2.3	0	S	-	-
Water-saturated butanol 73 g/L			S	-	-
n-hexane	1.88	0	SS	-	-
Chloroform	4.81	1.04 D	FS	-	-
Pyridine	12.4	2.37 D	FS	-	-
Dichloromethane	9.08	1.14 D	FS	-	-

**Table 6:** ADMET predicted profile for API Roseofungin (http://www.swissadme.ch, accessed 20 December 2024).

Model	Result	Probability
Absorption		
Blood–Brain Barrier	BBB−	0.5299
Human Intestinal Absorption	HIA+	0.589
Caco-2 Permeability	Caco2−	0.507
P-glycoprotein Substrate	Substrate	0.6851
P-glycoprotein Inhibitor	Non-inhibitor	0.8943
	Non-inhibitor	0.8116
Renal Organic Cation Transporter	Non-inhibitor	0.9083
Distribution		
Subcellular Localization	Mitochondria	0.7227
Metabolism		
CYP450 2C9 Substrate	Non-substrate	0.8097
CYP450 2D6 Substrate	Non-substrate	0.8794
CYP450 3A4 Substrate	Substrate	0.6131
CYP450 1A2 Inhibitor	Non-inhibitor	0.8395
CYP450 2C9 Inhibitor	Non-inhibitor	0.8426
CYP450 2D6 Inhibitor	Non-inhibitor	0.9166
CYP450 2C19 Inhibitor	Non-inhibitor	0.7379
CYP450 3A4 Inhibitor	Non-inhibitor	0.6126
CYP Inhibitory Promiscuity	Low CYP Inhibitory Promiscuity	0.9641
Human Ether-a-go-go-Related Gene Inhibition	Weak inhibitor	0.9063
	Non-inhibitor	0.9081
AMES Toxicity	Non-AMES Toxic	0.8658
Carcinogens	Non-carcinogens	0.9211
Fish Toxicity	High FHMT	0.7643
Tetrahymena Pyriformis Toxicity	High TPT	0.9306
Honeybee Toxicity	High HBT	0.7849
Biodegradation	Readily Biodegradable	0.7956
Acute Oral Toxicity	III	0.4429
Carcinogenicity (Three-class)	Non-required	0.7059

**Table 7:** ADMET predicted profile—regression for API *Roseofungin*.

Model	Value	Unit
Absorption		
Aqueous Solubility	−2.3794	LogS
Caco-2 Permeability	0.6006	LogPapp, cm/s
Rat Acute Toxicity	2.6839	LD50, mol/kg
Fish Toxicity	1.712	pLC50, mg/L
Tetrahymena Pyriformis Toxicity	0.3296	pIGC50, μg/L

**Table 8:** Comparative analysis of physico-chemical parameters of natural antifungal compounds.

Physico-Chemical Parameter	*Roseofungin*	*Nystatin*	*Amphotericin B*	*Natamycin*
Number of hydrogen bond acceptors	10	18	18	14
Number of hydrogen bond donors	6	12	12	7
Number of atoms	49	65	65	47
Topological polar surface area (TPSA), Å²	181.82	327	320 Å²	231
Octanol/water partition coefficient (logP)	5.4	0.5	0.8	1.1
Molecular weight, g/mol	690.9	926.1	924.1	665.7

## Data Availability

The original contributions presented in this study are included in the article. Further inquiries can be directed to the corresponding author.

## Supplementary Materials

The following supporting information can be downloaded at: https://www.mdpi.com/article/10.3390/pharmaceutics17040430/s1, Figure S1. Predicted ^1^H NMR spectrum of *Roseofungin* using Sci-Finder database. Figure S2. Results of macroscopic description of organs after evaluation of acute toxicity. After 14 days of observation, all the animals that received the *Roseofungin* test solutions were. Table S1. Changes in the body weight of mice after a single oral administration to mice of solutions of the substance *Roseofungin* in different dosages, (M ± m). Table S2. Weight of internal organs of mice after a single oral administration to mice of solutions of the substance *Roseofungin* in different dosages, (M ± m). Table S3. Weight coefficients of internal organs of mice after a single oral administration of solutions of *Roseofungin* substance to mice in different dosages, (M ± m).
