# Supplementary material for: Pharmaco-Technological Characterization, Structural Analysis, and Toxicological Evaluation of the Novel Polyene Antibiotic Roseofungin for Drug Development
Source: Pharmaceutics. 2025 Mar 27;17(4):430. doi: 10.3390/pharmaceutics17040430 (PMC12030180; doi:10.3390/pharmaceutics17040430)
Supplement: Supplementary file 1 [file pharmaceutics-17-00430-s001.zip › pharmaceutics-3419352-supplementary.pdf]

Supplementary information

PHARMACO-TECHNOLOGICAL CHARACTERIZATION, STRUCTURAL ANALYSIS, AND TOXICOLOGICAL EVALUATION OF THE NOVEL POLYENE ANTIBIOTIC ROSEOFUNGIN-AS FOR DRUG DEVELOPMENT

Sadanov Amankeldy[1,†], Berillo Dmitriy [2,3*,†], Bagimbayeva Assya[1], Baimakhanova Gul [1], Ibragimova Liliya [4*], Kulmaganbetov Iliyas [1], Nurmaganbetova Farida [1], Sarsenbaeva Gulbany [1], Orazymbet Saltanat [1], Baimakhanova Baiken [1], Lakh Olga Nikolaevna[1], Tleubekova Diana [1], Dzhakibaeva Gulnar [1] and Mussaldinov Tulegen [1]

[1] Scientific Production Center for Microbiology and Virology LLP, Almaty, Republic of Kazakhstan

[2] Department of chemistry, M. Kozybayev North-Kazakhstan University, Zhumabaeva str., 114, Petropavlovsk, 150000, Kazakhstan

[3] Department of pharmaceutical and toxicological chemistry, JSC "Asfendiyarov Kazakh National Medical University", Almaty, Kazakhstan

[4] Center for Pharmacy and Pharmacology of the Science and Technology Park, JSC Asfendiyarov Kazakh National Medical University, Almaty, Kazakhstan

Correspondence: berillo.d@kaznmu.kz , ibragimovaln19@gmail.com

† These authors contributed equally to this work.

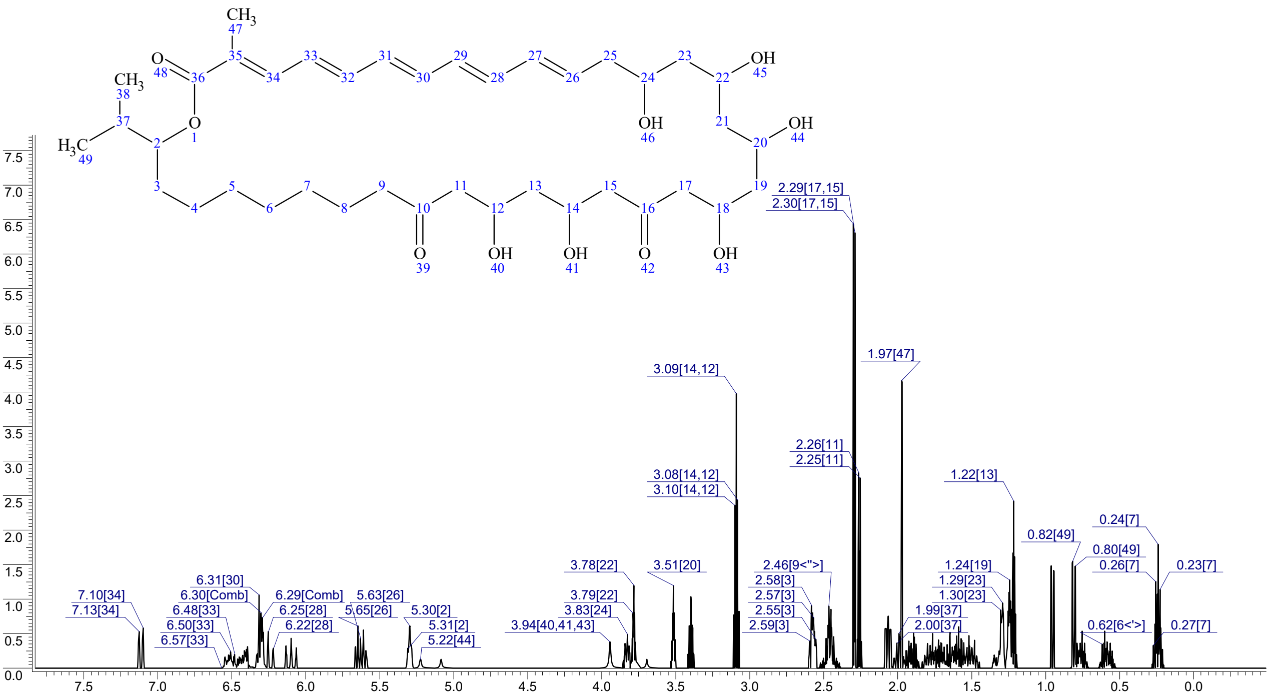

Group	nH	Shift	Error	Group	nH	Shift	Error	Group	nH	Shift	Error
2	1	5.30	0.48	14	1	3.09	0.30	30	1	6.30	0.17
3	2	2.57	0.29	15	2	2.29	0.33	31	1	6.30	0.17
4<">	1	1.91	0.15	17	2	2.29	0.33	32	1	6.31	0.23
4<'>	1	1.63	0.20	18	1	3.39	0.28	33	1	6.51	0.07
5<">	1	1.72	0.21	19	2	1.25	0.04	34	1	7.11	0.66
5<'>	1	1.57	0.27	20	1	3.51	0.10	37	1	1.99	0.11
6<">	1	0.77	0.20	21<">	1	1.33	0.10	38	3	0.95	0.03
6<'>	1	0.58	0.17	21<'>	1	1.25	0.05	40	1	3.94	1.11
7	2	0.24	0.79	22	1	3.78	0.08	41	1	3.94	1.11
8<">	1	1.78	0.21	23	2	1.29	0.23	43	1	3.94	1.11
8<'>	1	1.50	0.07	24	1	3.83	0.14	44	1	5.22	0.70
9<">	1	2.47	0.07	25	2	2.06	0.25	45	1	5.08	0.53
9<'>	1	2.44	0.06	26	1	5.63	0.09	46	1	3.69	0.08
11	2	2.26	0.33	27	1	6.10	0.06	47	3	1.97	0.07
12	1	3.09	0.30	28	1	6.25	0.09	49	3	0.81	0.03
13	2	1.22	0.10	29	1	6.42	0.52				

Figure S1. Predicted ^{1}H NMR spectrum of Roseofungin using Sci-Finder database.

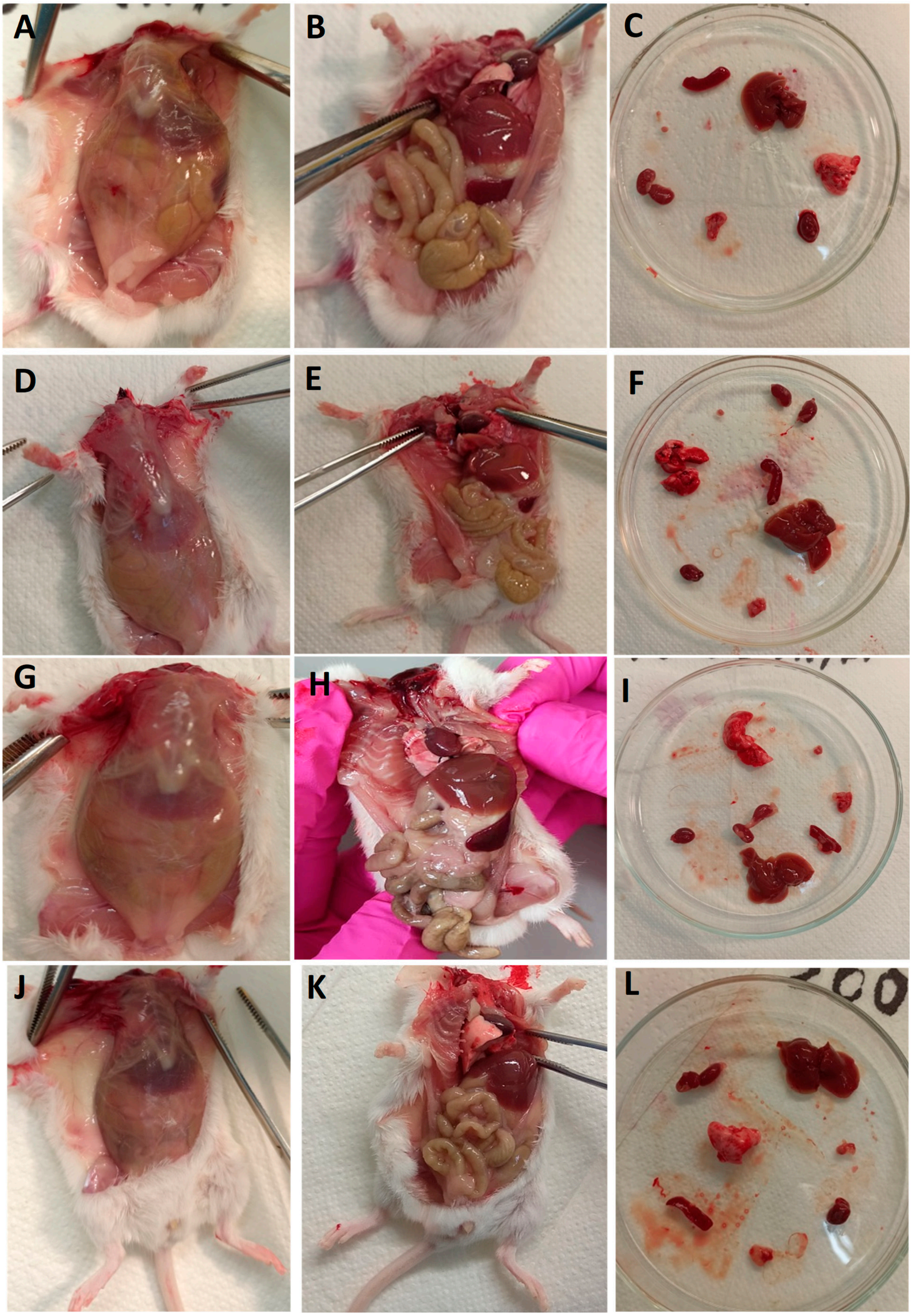

Figure S2. Results of macroscopic description of organs after evaluation of acute toxicity. After 14 days of observation, all the animals that received the *Roseofungin* test solutions were

euthanized by decapitation and macroscopically: A, B, C) 50 mg/kg ; D, E, F) 300 mg/kg ; G, H, I) 1000 mg/kg ; J, K, L) 2000 mg/kg .

Table S1. Changes in the body weight of mice after a single oral administration to mice of solutions of the substance Roseofungin in different dosages, (M ± m)

№ group	Dosage of the substance Roseofungina, mg/kg body weight	Average weight of animal, g		
		before experiment	after 1 week	after 2 weeks
1	50.0	19.76 ± 0.78	20.80 ± 2.36	22.80 ± 3.77
2	300.0	20.14 ± 0.67	22.02 ± 3.16	23.22 ± 2.99
3	1000.0	20.10 ± 0.57	21.02 ± 2.47	22.30 ± 1.69
4	2000.0	20.08 ± 0.64	20.10 ± 2.04	20.60 ± 2.13
Note:* – a reliable difference in relation to the values of body mass before the beginning of the experiment ($p < 0.05$)				

Table S2. Weight of internal organs of mice after a single oral administration to mice of solutions of the substance Roseofungin in different dosages, (M ± m)

№ group	Dosage of roseofungin, mg/kg body weight		Weight of internal organs of mice at the moment of slaughter (after 14 days of observation), g						
			Liver	Lung	Spleen	Kidneys	Heart	Thymus	Adrenal glands
1	50.0	M	1.5674	0.2194	0.1173	0.2565	0.1046	0.0566	0.0112
		m	0.1120	0.0527	0.0258	0.0291	0.0091	0.0096	0.0033
2	300.0	M	1.5028	0.2678	0.1206	0.2662	0.1093	0.0533	0.0083
		m	0.1487	0.0416	0.0227	0.0355	0.0074	0.0057	0.0013
3	1000.0	M	1.5673	0.2317	0.1046	0.2527	0.1078	0.0487	0.0083
		m	0.0577	0.0353	0.0089	0.0179	0.0066	0.0067	0.0004
4	2000.0	M	1.5219	0.2199	0.0906	0.2269	0.0986	0.0438	0.0112
		m	0.1180	0.0493	0.0014	0.0078	0.0026	0.0083	0.0044

Table S3. Weight coefficients of internal organs of mice after a single oral administration of solutions of *Roseofungin* substance to mice in different dosages, (M ± m)

№	Dosage of roseofungin, mg/kg body weight		Weight coefficients of internal organs of mice at the time of slaughter (after 14 days of observation), %						
			Liver	Lung	Spleen	Kidneys	Heart	Thym us	Adrenal glands
1	50.0	M	6.985	0.973	0.516	1.143	0.467	0.252	0.050
		m	0.9131	0.2226	0.0778	0.1833	0.0709	0.0535	0.0174
2	300.0	M	6.554	1.154	0.520	1.154	0.477	0.230	0.036
		m	0.9611	0.1225	0.0715	0.1484	0.0662	0.0144	0.0056
3	1000.0	M	7.066	1.036	0.471	1.137	0.485	0.218	0.038
		m	0.6726	0.0906	0.0530	0.1036	0.0426	0.0249	0.0037
4	2000.0	M	6.804	0.973	0.403	1.008	0.439	0.193	0.051
		m	1.0176	0.2120	0.0319	0.0580	0.0377	0.0272	0.0237
